# Supplementary material for: New Antineoplastic Naphthohydroquinones Attached to Labdane and Rearranged Diterpene Skeletons
Source: Molecules. 2021 Jan 18;26(2):474. doi: 10.3390/molecules26020474 (PMC7831060; doi:10.3390/molecules26020474)
Supplement: Supplementary file 1 [file molecules-26-00474-s001.pdf]

Supplementary Materials

New antineoplastic naphthohydroquinones attached to labdane and rearranged diterpene skeletons

Ángela P. Hernández[1,2], Pablo Chamorro[1], Mª Lucena Rodríguez[1], José Mª Miguel del Corral[1], Pablo A. García[1], Andrés Francesch[3], Arturo San Feliciano[1,4], Mª Ángeles Castro[1,*]

[1] Departamento de Ciencias Farmacéuticas, Área de Química Farmacéutica, Facultad de Farmacia, CIETUS/IBSAL, University of Salamanca, Campus Miguel de Unamuno, 37007 Salamanca, Spain; angytahg@usal.es (Á.P.H.); chamorrosanchez@gmail.com (PC); mlucenarodriguez@gmail.com (M.L.R.); jmmcs@usal.es (J.M.M.C.); pabloagg@usal.es (P.A.G.); artsf@usal.es (A.S.F.)

[2] Department of Medicine and General Cytometry Service-Nucleus, CIBERONC CB16/12/00400, Cancer Research Centre (IBMCC/CSIC/USAL/IBSAL), 37007 Salamanca, Spain.

[3] PharmaMar S.A., Avda de los Reyes, 1; 28770 Colmenar Viejo, Madrid, Spain; afrancesch@pharmamar.com

[4] Universidade do Vale do Itajaí, Programa de Pós-graduaçao em Ciências Farmacêuticas, UNIVALI. 88302-202 Itajaí, SC, Brazil

[*] Correspondence: macg@usal.es. Tel.: +34 923294500 ext: 1824

Table S1. Correlations and assignments for compound **8** (δ in ppm).

Table S2. Correlations and assignments for compound **9a** (δ in ppm).

Table S3. Correlations and assignments for compound **10** (δ in ppm).

Table S4. Correlations and assignments for compound **11** (δ in ppm).

Table S5. Correlations and assignments for compound **14** (δ in ppm).

Table S6. Correlations and assignments for compound **15** (δ in ppm).

Table S7. Correlations and assignments for compound **19** (δ in ppm).

Figure S1: ^{1}H and ^{13}C NMR spectra for compound **3**.

Figure S2: ^{1}H and ^{13}C NMR spectra for compound **5.**

Figure S3: ^{1}H and ^{13}C NMR spectra for compound **6.**

Figure S4: ^{1}H and ^{13}C NMR spectra for compound **7.**

Figure S5:^{1}H and ^{13}C NMR spectra for compound **8**.

Figure S6: HMQC and HMBC experiments for compound **8**.

Figure S7: IR, ^{1}H and ^{13}C NMR spectra for compound **9a**.

Figure S8: HMQC and HMBC experiments for compound **9a**.

Figure S9: ^{1}H and ^{13}C NMR spectra for compound **10**.

Figure S10: HMQC and HMBC experiments for compound **10**

Figure S11: ^{1}H and ^{13}C NMR spectra for compound **11**.

Figure S12:^{1}H and ^{13}C NMR spectra for compound **12**.

Figure S13: ^{1}H and ^{13}C NMR spectra for compound **13**.

Figure S14:^{1}H and ^{13}C NMR spectra for compound **14**.

Figure S15: HMQC and HMBC experiments for compound **14.**

Figure S16: ^{1}H and ^{13}C NMR spectra for compound **15**.

Figure S17: HMQC and HMBC experiments for compound **15.**

Figure S18: ^{1}H and ^{13}C NMR spectra for compound **16a**.
Figure S19: ^{1}H and ^{13}C NMR spectra for compounds **16a-c**.
Figure S20: ^{1}H and ^{13}C NMR spectra for compound **17**.
Figure S21: ^{1}H and ^{13}C NMR spectra for compound **18a**.
Figure S22: ^{1}H and ^{13}C NMR spectra for compounds **18b** and **18c**.
Figure S23: ^{1}H and ^{13}C NMR spectra for compound **18c**.
Figure S24: ^{1}H and ^{13}C NMR spectra for compounds **19**.
Figure S25: HMQC and HMBC experiments for compound **19**.
Figure S26: IR, ^{1}H and ^{13}C NMR spectra for compounds **20**.
Figure S27: ^{1}H and ^{13}C NMR spectra for compounds **21a**.
Figure S28: 1H and 13C NMR spectra for compounds **21c**.

Table S1. Correlations and assignments for compound **8** (δ in ppm).

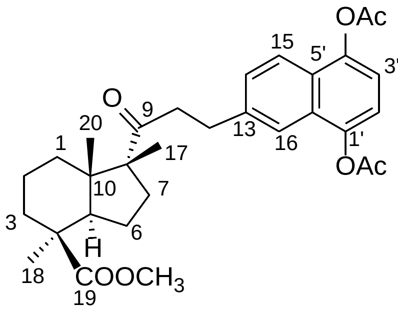

δ ^{13}C	TYPE*	HMQC, δ ^{1}H (J in Hz)	HMBC, δ ^{1}H (H number)	Assigned
35.1	CH_2	1.55 *m*, 0.65 *m*	0.65 (20)	1
36.8	CH_2	2.06 *m*, 0.65 *m*	1.15 (18)	3
44.0	C	--	1.15 (18)	4
52.9	CH	1.65 *m*	0.65 (20), 1.15 (18)	5
23.1	CH_2	2.10 *m*, 1.88 *m*	1.65 (5)	6
33.2	CH_2	1.32 *m*	1.17 (17)	7
60.6	C	--	1.17 (17), 0.65 (20)	8
216.1	C	--	1.17 (17), 2.97 (12b) 2.79 (11)	9
47.0	C	--	0.65 (20), 1.17 (17)	10
43.2	CH_2	2.79 *m*	--	11
30.4	CH_2	3.09 *m*, 2.97 *m*	7.63 (16)	12
140.5	C	--	7.77 (15)	13
128.4	CH	7.38 *d* (8.6 Hz)	7.63 (16)	14
121.8	CH	7.77 *d* (8.6 Hz)	--	15
120.4	CH	7.63 *s*	7.38 (14)	16
20.1	CH_3	1.17 *s*	--	17
28.3	CH_3	1.15 *s*	--	18
178.1	C	--	3.63 (COO<u>CH$_3$</u>)<br>1.15 (18)	19
14.8	CH_3	0.65 *s*	--	20
143.9	C	--	7.63 (16), 7.17 (2’)	1’
116.9	CH	7.21 *d* (8.2 Hz)	--	2’
117.8	CH	7.17 *d* (8.2 Hz)	--	3’
144.3	C	--	7.77 (15), 7.21 (2’)	4’
126.2	C	--	7.63 (16), 7.17 (3’), 7.38 (14)	5’
127.7	C	--	7.21 (2’), 7.77 (15)	6’
169.3 169.2	C	--	2.48, 2.44 [OAc (2xCH_3)]	OAc (2xCO)
21.0	CH_3	2.44 *s*, 2.48 *s*	--	OAc (2xCH_3)
51.1	CH_3	3.63 *s*	--	COO<u>CH$_3$</u>

* Carbon type according to DEPT experiment

Table S2. Correlations and assignments for compound **9a** (δ in ppm).

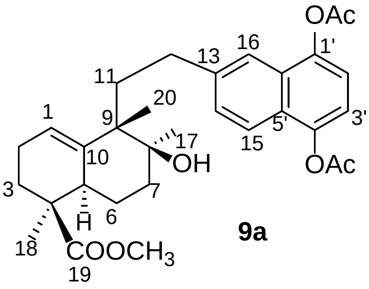

9a

δ ^{13}C	TYPE*	HMQC, δ ^{1}H (*J* in Hz)	HMBC, δ ^{1}H (H number)	Assigned
120.5	CH	5.52 m	--	1
22.3	CH_2, CH_3	1.37 s	--	2, 18
24.7	CH_2	--	1.37 (18)	3
44.4	C	--	1.37 (18)	4
41.4	CH	2.15 m	1.37 (18)	5
37.3	CH_2	1.55 m	1.13 (17)	7
75.9	C	--	1.13 (17)	8
48.5	C	--	1.15 (20)	9
140.9	C	--	1.15 (20)	10
35.7	CH_2	2.11 m	1.15 (20)	11
30.9	CH_2	2.46 m	7.59 (16), 7,39 (14)	12
142.3	C	--	7.78 (15)	13
128.5	CH	.39 *dd* (8.8, 1.6 Hz	7.59 (16)	14
121.7	CH	7.78 *d* (8.8 Hz)	--	15
119.8	CH	7.59 *bs*	7.39 (14)	16
24.0	CH_3	1.13 *s*	--	17 or 20
117.7	C	--	3.70 (COO<u>CH</u>$_3$)	19
16.8	CH_3	1.15 *s*	1.37 (18)	20 or 17
143.8	C	--		1’
116.7	CH	7.16 *d* (8.4 Hz)	7.59 (16), 7.21 (3’)	2’
117.7	CH	7.21 *d* (8.4 Hz)	--	3’
144.3	C	--	--	4’
125.2	C	--	7.78 (15), 7.16 (2’)	5’
127.8	C	--	7.59 16, 7.21 (3’), 7.39 (14)	6’
169.4	C	--	2.48, 2.44 [OAc (2xCH_3)]	OAc (2xCO)
21.0	CH_3	2.45 *s*, 2.46 *s*	--	OAc (2xCH_3)
51.5	CH_3	3.70 *s*	--	COO<u>CH</u>$_3$

* Carbon type according to DEPT experiment

Table S3. Correlations and assignments for compound **10** (δ in ppm).

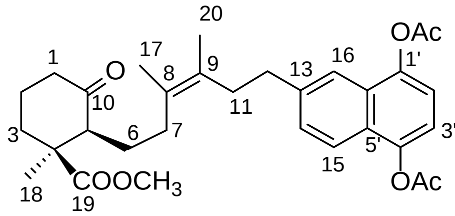

δ ¹³C	TYPE*	HMQC, δ ¹H (*J* in Hz)	HMBC, δ ¹H (H number)	Assigned
18.3	CH_3	1.68 s	-	17
18.5	CH_3	1.70 s	-	20
21.0	CH_3	2.44 s; 2.48 s	-	OAc (Me)
24.2	CH_3	1.28 s	-	18
33.3	CH_2	-	1.68 (17)	7
33.8	CH_2	-	1.28 (18)	3
35.4	CH_2	2.79 m	7.42 (14), 7.63 (16)	12
36.2	CH_2	-	1.70 (20), 2.79 (12)	11
50.4	C	-	1.28 (18)	4
51.7	CH_3	3.61 s	-	OMe
58.0	CH	-	1.28 (18)	5
116.6	CH	7.20 d	-	3´
117.6	CH	7.16 d	-	2´
119.9	CH	7.63 bs	7.42 (14), 2.79 (12)	16
121.5	CH	7.78 d	-	15
126.1	C	-	7.20 (3´), 7.63 (16), 7.42 (14)	5´
127.8	C	-	1.70 (20)	8
127.8	C	-	7.16 (2´), 7.78 (15)	6´
127.9	C	-	1.68 (17), 2.79 (12)	9
128.5	CH	7.42 dd	7.63 (16), 2.79 (12)	14
141.5	C	-	7.78 (15), 2.79 (12)	13
144.0	C	-	7.20 (3´), 7.63 (16)	1´
144.3	C	-	7.16 (2´), 7.78 (15)	4´
169.2;169.4	C	-	2.44;2.48 (OAc-Me)	OAc (CO)
175.5	C	-	1.28 (18), 3.61 (OMe)	19

* Carbon type according to DEPT experiment

Table S4. Correlations and assignments for compound **11** (δ in ppm).

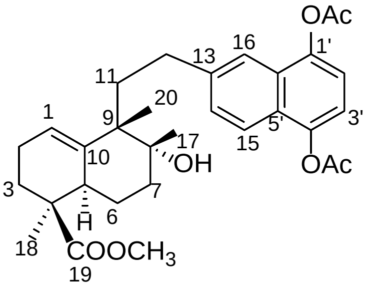

δ ^{13}C	TYPE*	HMQC, δ ^{1}H (*J* in Hz)	HMBC, δ ^{1}H (H number)	Assigned
122.4	CH	5.61 m	2.25 (5)	1
24.3	CH_2	--	1.40 (18)	3
44.4	C	--	1.40 (18)	4
41.4	CH	2.25 m	1.40 (18)	5
27.4	CH_2	1.39 m	--	6 or 2
35.4	CH_2	1.71 m, 1.52 m	1.11 (17)	7
75.0	C	--	1.11 (17), 1.19 (20)	8
49.0	C	--	1.11 (17), 1.19 (20)	9
140.8	C	--	1.64 (11b), 1.19 (20)	10
38.0	CH_2	2.09 m, 1.64 m	1.19 (20)	11
31.4	CH_2	2.64 m, 2.43 m	7.59 (16), 7.39 (14)	12
141.8	C	--	6.62 (12a), 2.43 (12b), 7.80 (15)	13
128.2	CH	7.39 *dd* (8.6, 1.6 Hz)	6.62 (12a), 2.43 (12b), 7.59 (16)	14
121.9	CH	7.80 *d* (8.6 Hz)	--	15
119.8	CH	7.59 *bs*	6.62 (12a), 2.43 (12b), 7.39 (14)	16
17.2	CH_3	1.11 *s*	--	17
22.4	CH, CH_3	1.40 *s*	--	2 or 6, 18
177.5	C	--	1.40 (18), 3.72 (COO<u>CH_3</u>)	19
22.7	CH_3	1.19 *s*	--	20
143.9	C	--	7.59 (16), 7.18 (3’)	1´
116.8	CH	7.23 *d* (8.2 Hz)	--	2´
117.8	CH	7.21 *d* (8.2 Hz)	--	3´
144.3	C	--	7.80 (15), 7.23 (2’)	4´
126.1	C	--	7.59 (16), 7.39 (14), 7.18 (3’)	5´
127.8	C	--	7.23 (2’), 7.80 (15)	6´
169.3	C	--	2.47, 2.45 [OAc (2xCH_3)]	OAc (2x CO)
20.9	CH_3	2.47 *s*, 2.45 *s*	--	OAc (2xCH_3)
51.5	CH_3	3.72 *s*	--	OCH_3

* Carbon type according to DEPT experiment

Table S5. Correlations and assignments for compound **14** (δ in ppm).

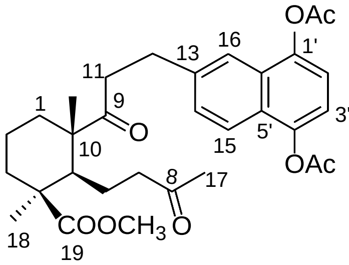

δ ^{13}C	TYPE*	HMQC, δ ^{1}H (*J* in Hz)	HMBC, δ ^{1}H (H number)	Assigned
18.8	CH_2,CH_3	56 m, 1.11 m, 1.0(	--	2, 20
34.5	CH_2	8 m, 1.61 m, 1.25	1.12 (18), 1.00 (20)	1, 3
48.0	CH	1.99 m	1.12 (18), 1.00 (20)	5
23.3	CH_2	--	2.47 (7b)	6
46.1	C, CH_2	2.51 m, 2.47 m	1.99 (5), 1.12 (18)	4,7
208.5	C	--	2.04 (17), 2.47 (7b)	8
214.7	C	--	1.00 (20); 3.03, 2.89 (11/12)	9
52.6	C	3.03 m, 2.89 m	1.00 (20)	10
39.9	CH_2	3.03 m, 2.89 m	3.03 (12a)	11
30.5	CH_2	--	7.63 (16), 7.39 (14), 2.89 (11b)	12
140.5	C	.39 *dd* (8.8, 1.3 H	7.78 (15); 3.03, 2.89 (11/12)	13
128.3	CH	7.78 *d* (8.8 Hz)	7.63 (16), 3.03 (12a)	14
122.0	CH	7.63 *bs*	--	15
120.4	CH	2.04 *s*	7.39 (14), 3.03 (12a	16
29.8	CH_3	1.12 *s*	--	17
27.0	CH_3		--	18
177.7	C		3.64 (COO$\underline{CH_3}$), 1.12 (18)	19
144.0	C		7.63 (16)	1´
117.1	CH	7.21 *d* (8.3 Hz)	--	2´
118.0	CH	7.16 *d* (8.3 Hz)	--	3´
144.4	C	--	7.78 (15)	4´
126.3	C		7.63 (16), 7.39 (14), 7.16 (3’)	5´
127.8	C		7.78 (15); 7.21 (2’)	6´
169.5	C		2.47, 2.44 [OAc (2x CH_3)]	OAc (2xCO)
21.1	CH_3	2.47 *s*, 2.44 *s*		OAc (2xCH_3)
51.6	CH_3	3.64 *s*		OCH_3

* Carbon type according to DEPT experiment

Table S6. Correlations and assignments for compound **15** (δ in ppm).

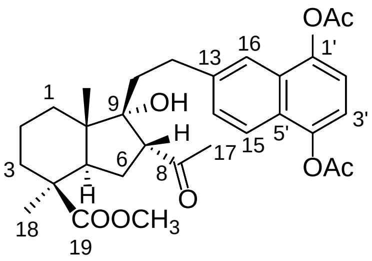

δ ^{13}C	TYPE*	HMQC, δ ^{1}H (*J* in Hz)	HMBC, δ ^{1}H (H number)	Assigned
30.4	CH_2	1.37 *m*, 1.37 *m*	0.70 (20)	1
19.9	CH_2	1.57 *m*, 184 *m*	1.37 (1b)	2
37.2	CH_2	1,01 *m*, 2.21 *m*	1.20 (18), 1.37 (1b)	3
43.9	C	--	1.20 (18), 1.57 (2b), 2,32 (5)	4
51.8	CH	2.32 *dd* (8.7, 11.9 Hz)	0.70 (20), 1.20 (18), 1.37 (1b)	5
27.9	CH_2	1.81 *m*, 2.76 *m*	2.32 (5), 2.96 (7)	6
53.2	CH	2.96 *m*	2.22 (17), 5.34 (OH)	7
216.1	C	--	1.81 (6b), 2.22 (17), 2.96 (7)	8
84.3	C	--	0.70 (20), 5.34 (OH)	9
49.2	C	--	7 (1b), 2.32 (5), 0.70 (20), 5.34 (C	10
38.8	CH_2	1.87 *m*	--	11
31.3	CH_2	2.66 *m*, 2,92 *m*	1.87 (11), 7.36 (14), 7.60 (16)	12
141.6	C	--	2.92 (12a), 2.66 (12b), 7.76 (15)	13
128.3	CH	7.36 *dd* (8.6, 1.4 Hz)	7.60 (16)	14
121.7	CH	7.78 *d* (8.6 Hz)	7.36 (14)	15
119.9	CH	7.60 *bs*	7.36 (14)	16
31.5	CH_3	2.22 *s*	--	17
28.2	CH_3	1.20 *s*	1.01 (3b), 1.37 (1b),	18
177.8	C	--	.20 (18), 3.68 ($COOCH_3$), 2.32 (5	19
14.9	CH_3	0.70 *s*	1.37 (1b), 2.32 (5)	20
143.9	C	--	7.20 (2’) 7.76 (15)	1´
116.7	CH	7.20 *d* (8.3 Hz)	7.15 (3’)	2´
117.7	CH	7.15 *d* (8.3 Hz)	7.20 (2’)	3´
144.3	C	--	7.20 (2’) 7.76 (15)	4´
126.1	C	--	7.15 (3’), 7.36 (14), 7.60 (16)	5´
127.8	C	--	7.20 (2’) 7.76 (15)	6´
169.3,169.2	C	--	2.44, 2.47 [OAc (2xCH_3)]	OAc (2xCO)
20.9,21.0	CH_3	2.47 *s*, 2.44 *s*		OAc (2xCH_3)
51.3	CH_3	3.68 *s*		OCH_3

* Carbon type according to DEPT experiment

Table S7. Correlations and assignments for compound **19** (δ in ppm).

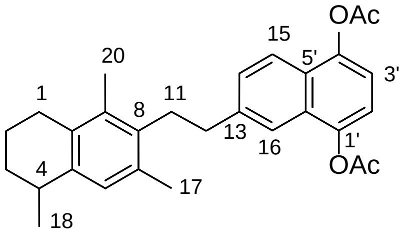

δ ^{13}C	TYPE*	HMQC, δ ^{1}H (*J* in Hz)	HMBC, δ ^{1}H (H number)	Assigned
27.8	CH_2	2.62 *m*	--	1
20.4	CH_2	2.62 *m*	--	2
30.7	CH_2	1.91 *m*	1.30 (18)	3
32.7	CH	--	6.94 (6), 1.30 (18)	4
139.9	C	--	1.30 (18)	5
127.9	CH	6.94 *bs*	2.35 (17)	6
132.9	C	--	2.35 (17)	7
135.2	C	--	6.94 (6), 2.35 (17)	8
134.2	C	--	2.23 (20)	9
133.2	C	--	6.94 (6), 2.23 (20)	10
31.9	CH_2	2.89-3.01 *m*	2.89-3.01 (12)	11
36.0	CH_2	2.89-3.01 *m*	2.89-3.01 (11), 7.65 (16, 7.47 (14)	12
141.3	C	--	2.89-3.01 (11,12) 7.82 (15)	13
128.3	CH	7.47 *d* (8.6 Hz)	7.65 (16)	14
121.8	CH	7.82 *d* (8.6 Hz)	--	15
119.7	CH	7.65 *bs*	7.47 (14)	16
20.0	CH_3	2.35 *s*	6.94 (6)	17
23.0	CH_3	1.30 *d* (7.0 Hz)	--	18
15.0	CH_3	2.23 *s*	--	20
143.9	C	--	7.65 (16), 7.19 (3’)	1´
116.8	CH	7.22 *d* (8.2 Hz)	--	2´
117.7	CH	7.17 *d* (8.2 Hz)	--	3´
144.3	C	--	7.82 (15), 7.22 (2’)	4´
126.2	C	--	7.65 (16), 7.47 (14), 7.19 (3’)	5´
127.9	C	--	7.22 (2’), 7.82 (15)	6´
169.3	C	--	2.47, 2.47 [(OAc (2x CH_3)]	OAc (2xCO)
20.9	CH_3	2.46 *s*, 2.47 *s*	--	OAc (2x CH_3)

* Carbon type according to DEPT experiment

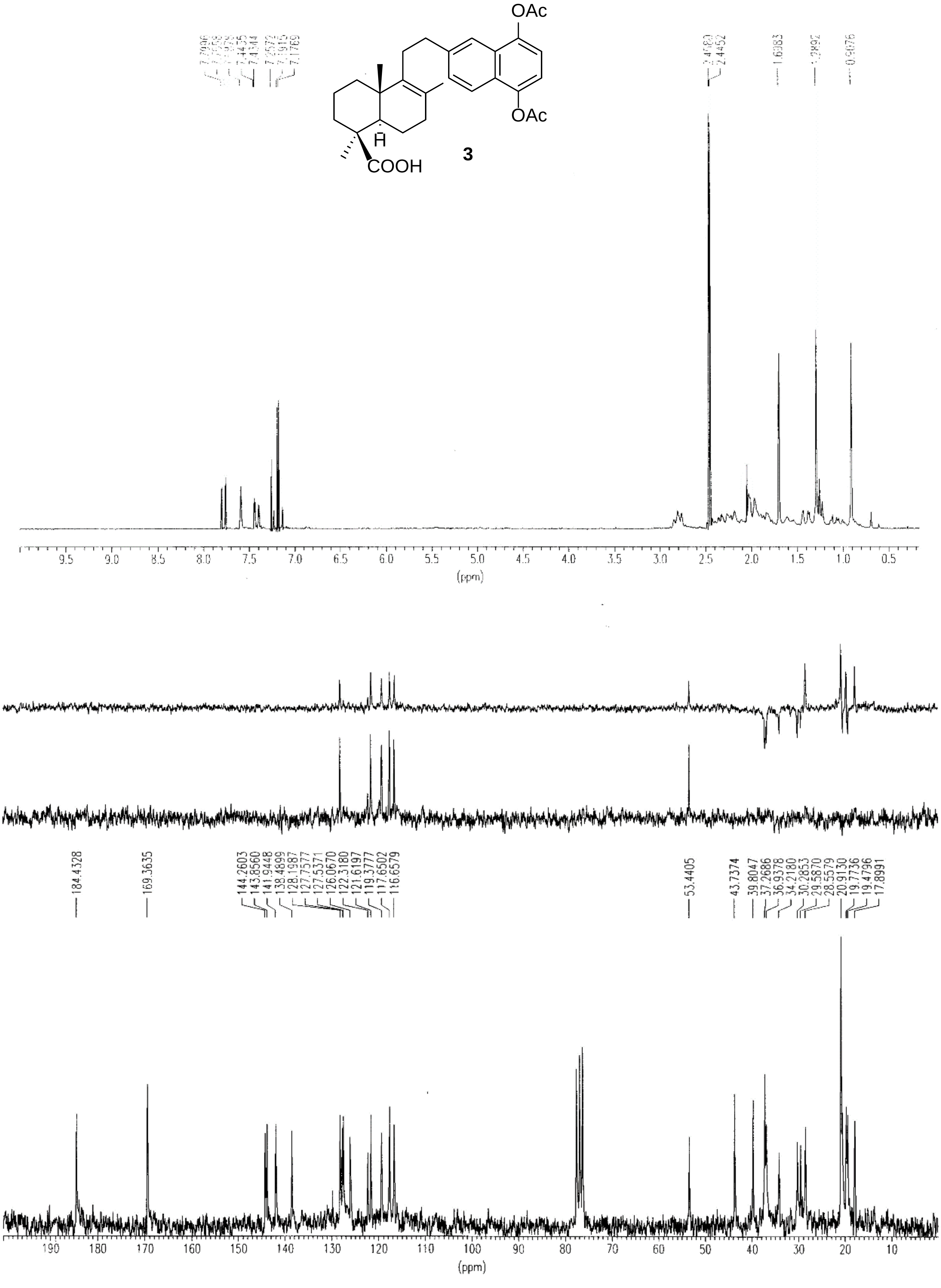

Figure S1: ^{1}H and ^{13}C NMR spectra for compound **3**.

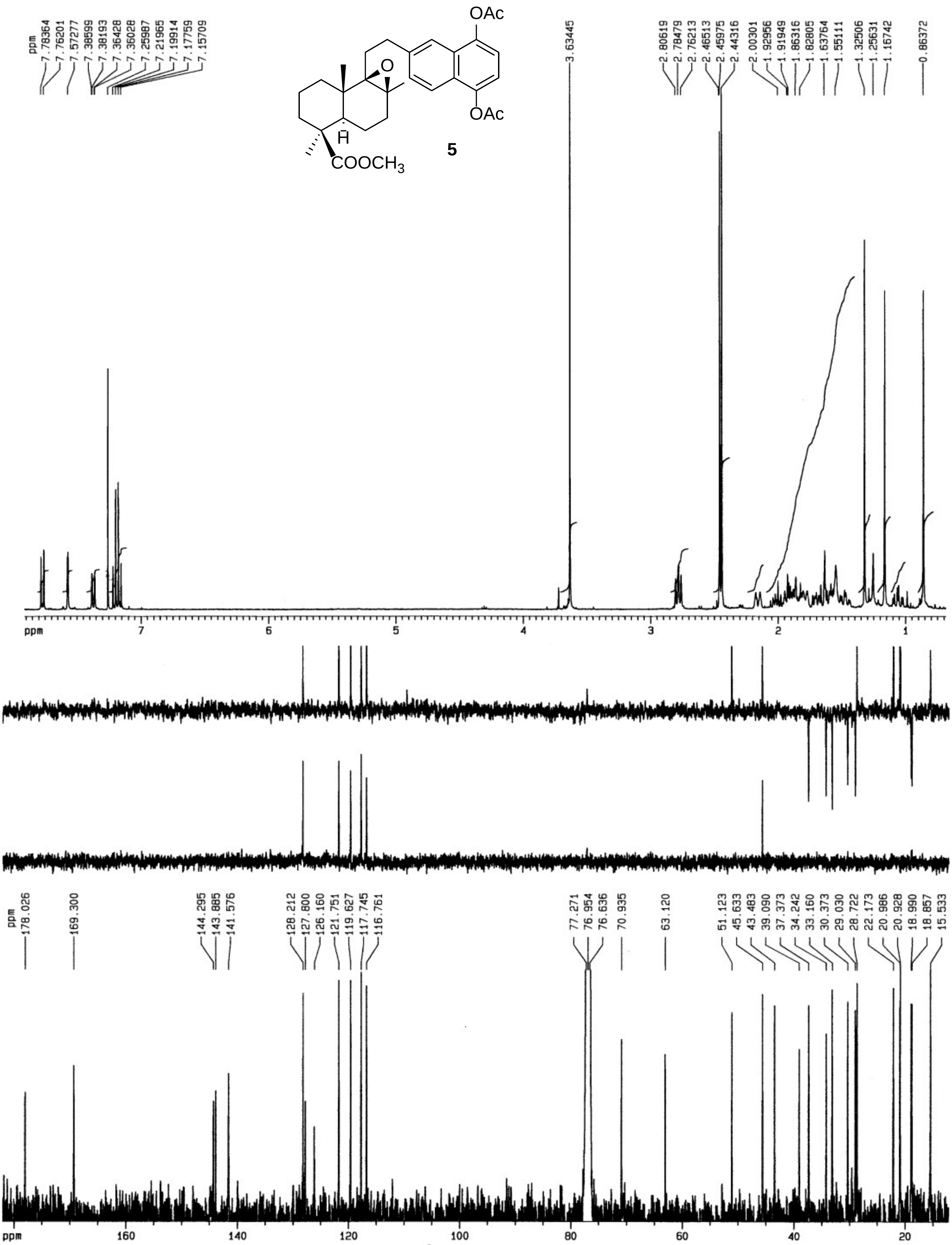

Figure S2: ^{1}H and ^{13}C NMR spectra for compound **5.**

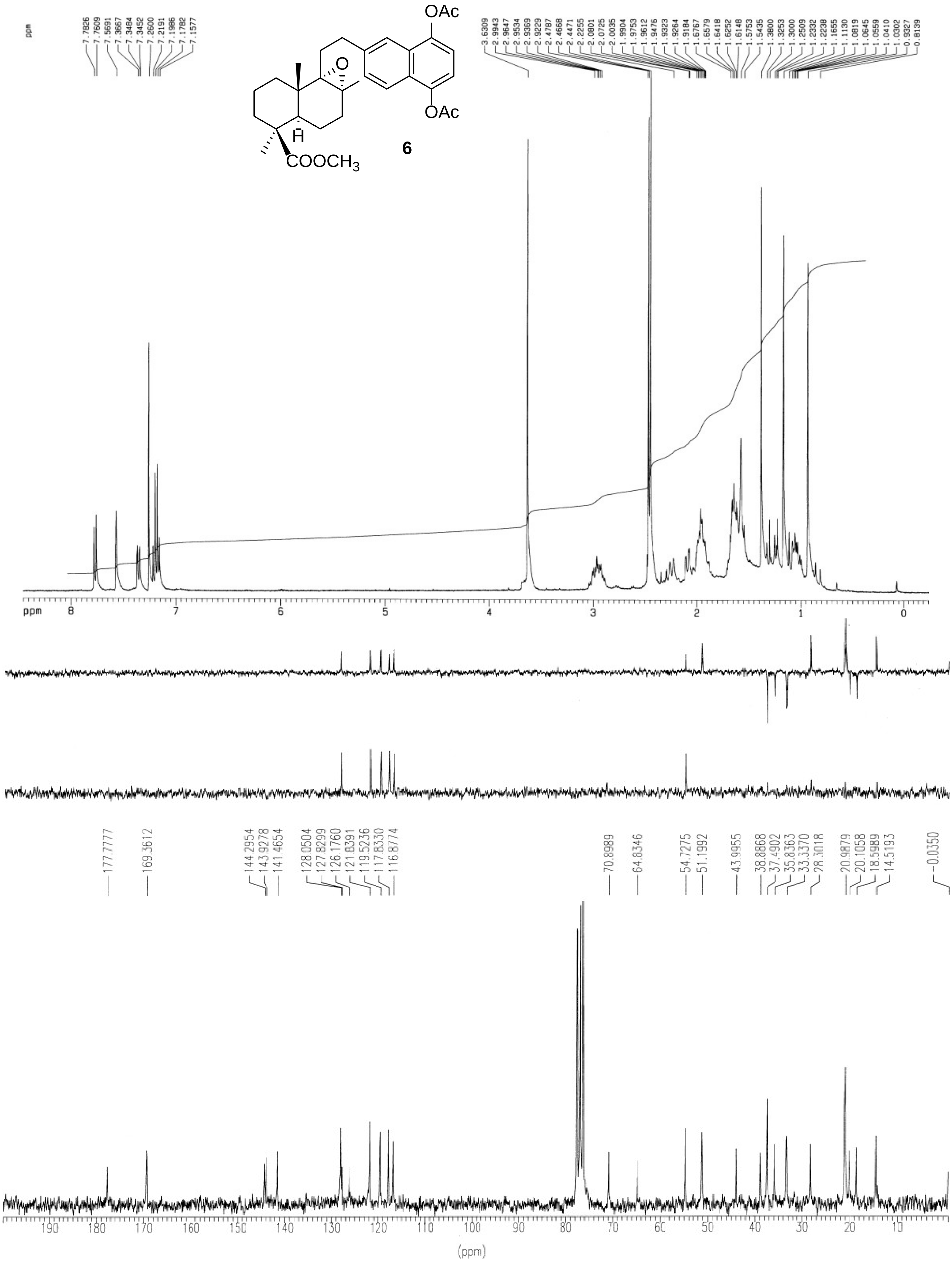

Figure S3: ^{1}H and ^{13}C NMR spectra for compound **6.**

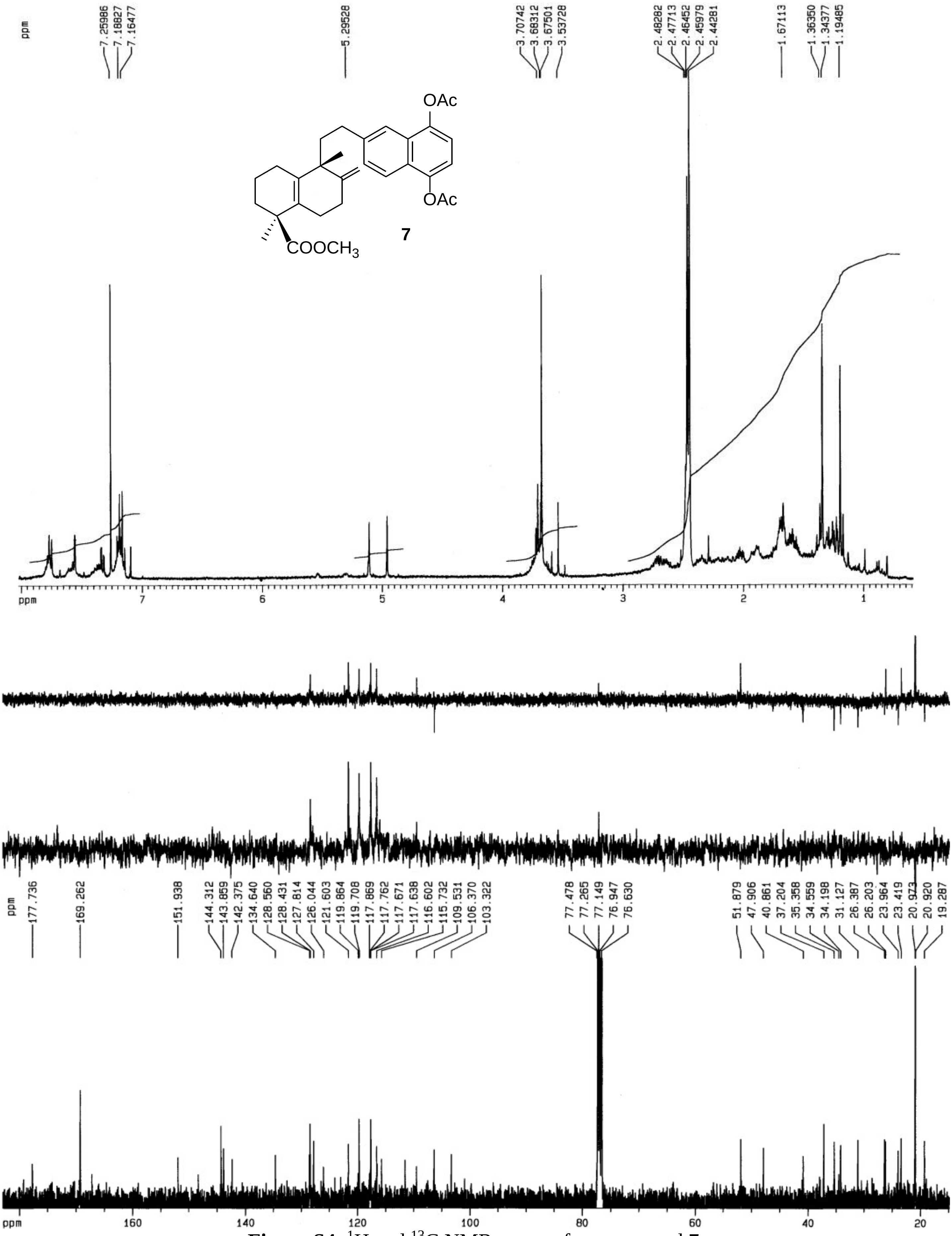

Figure S4: ^{1}H and ^{13}C NMR spectra for compound **7.**

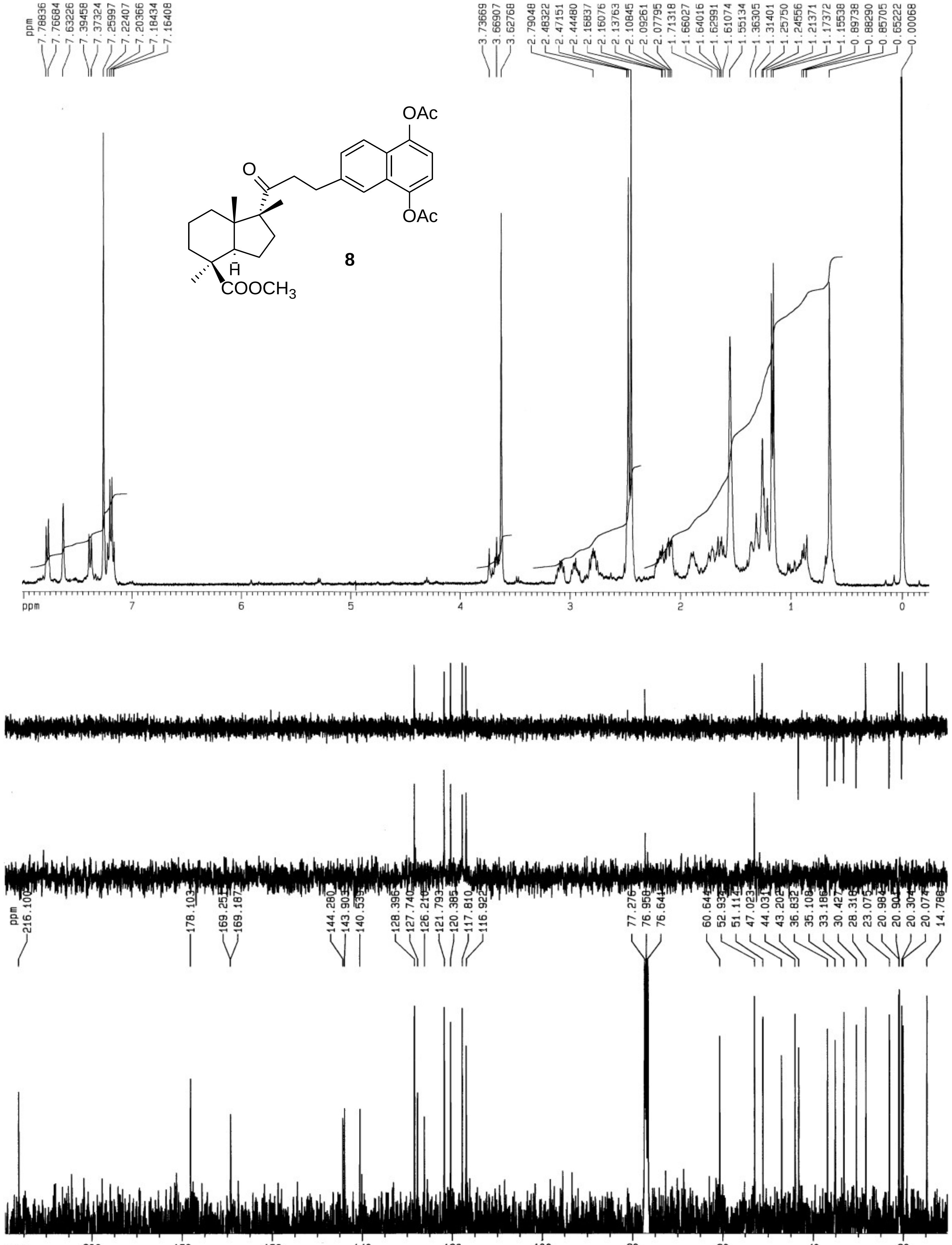

Figure S5:^{1}H and ^{13}C NMR spectra for compound **8**.

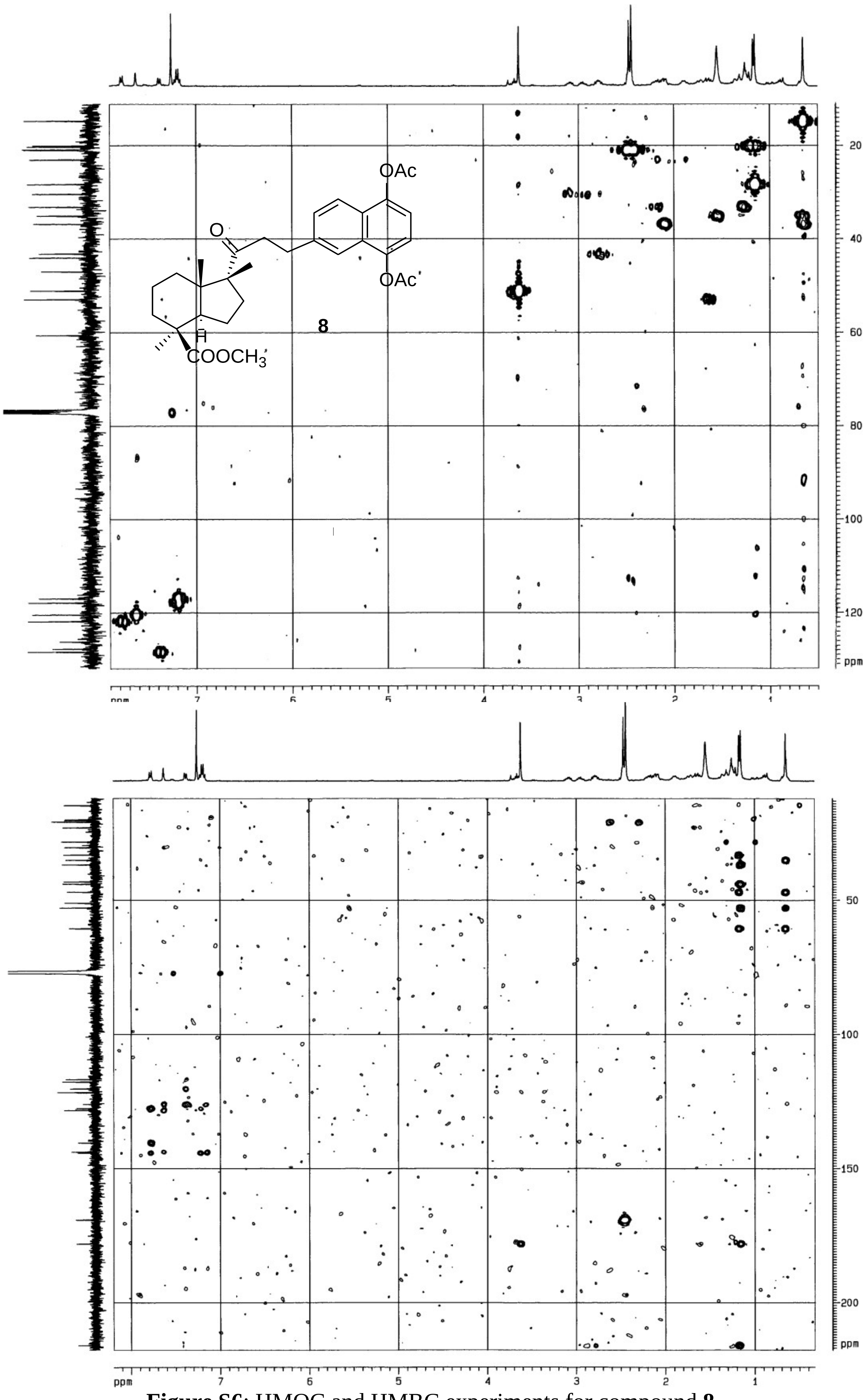

Figure S6: HMQC and HMBC experiments for compound **8**.

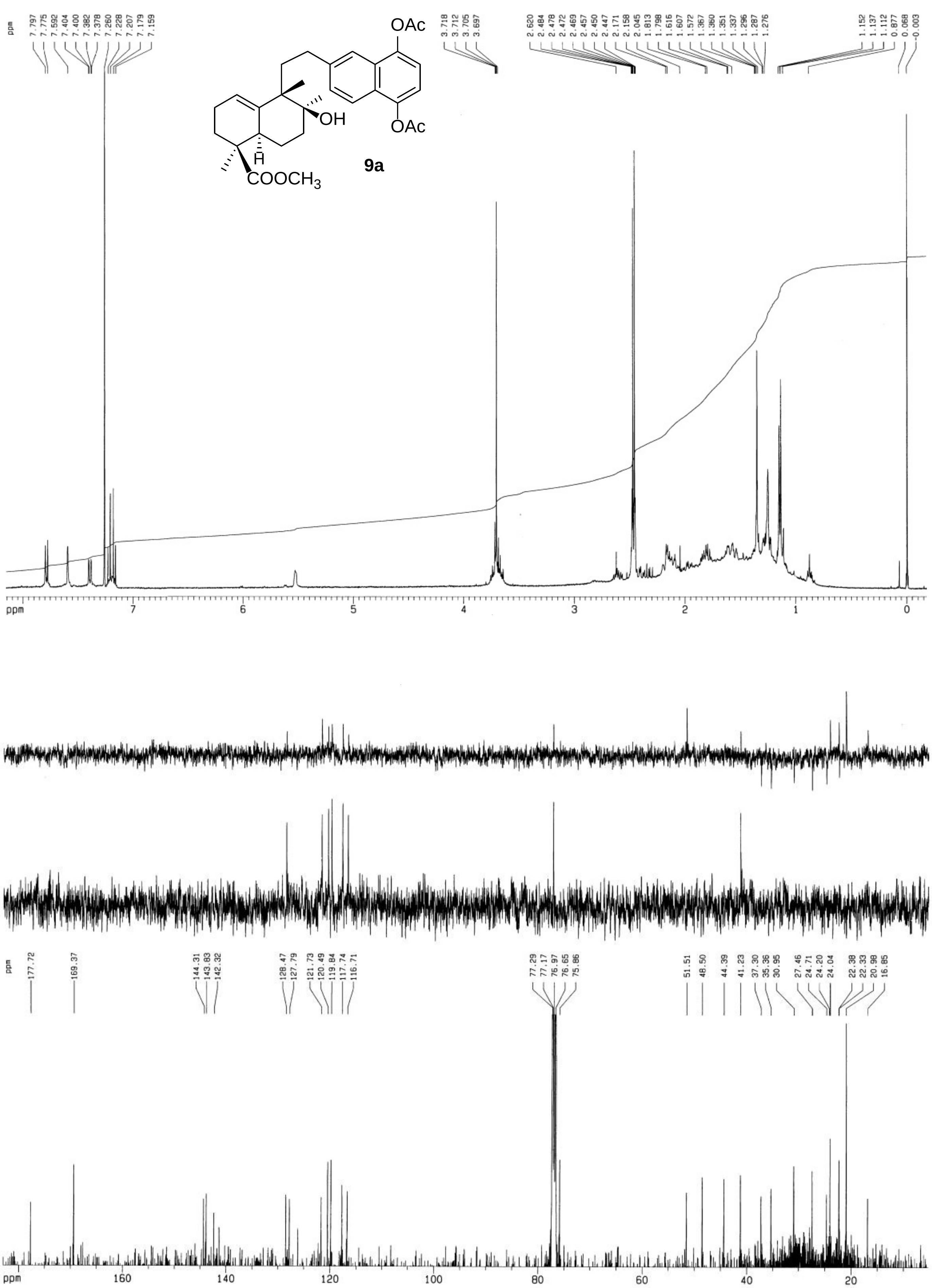

Figure S7: IR, ^{1}H and ^{13}C NMR spectra for compound **9a**.

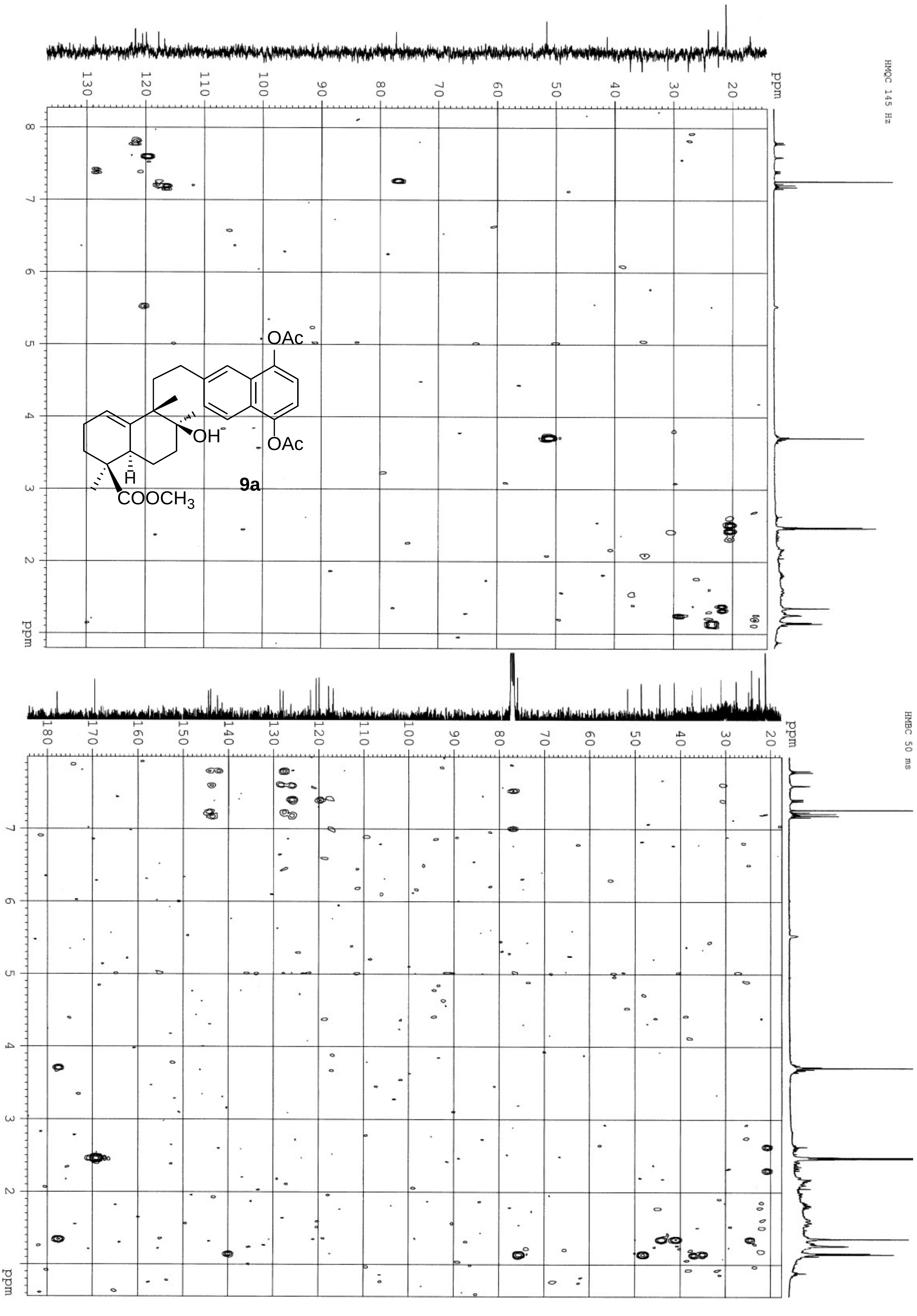

Figure S8: HMQC and HMBC experiments for compound **9a**.

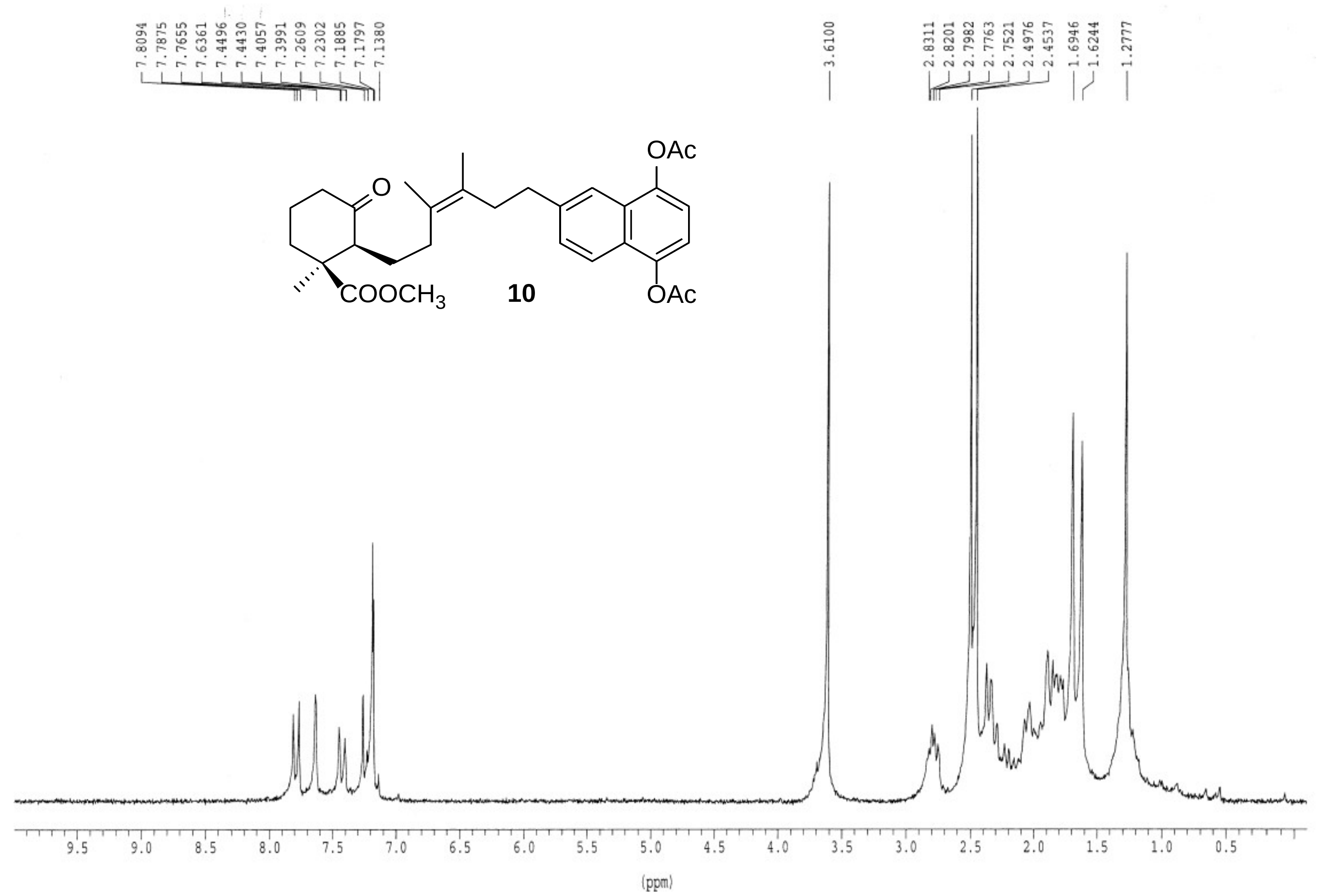

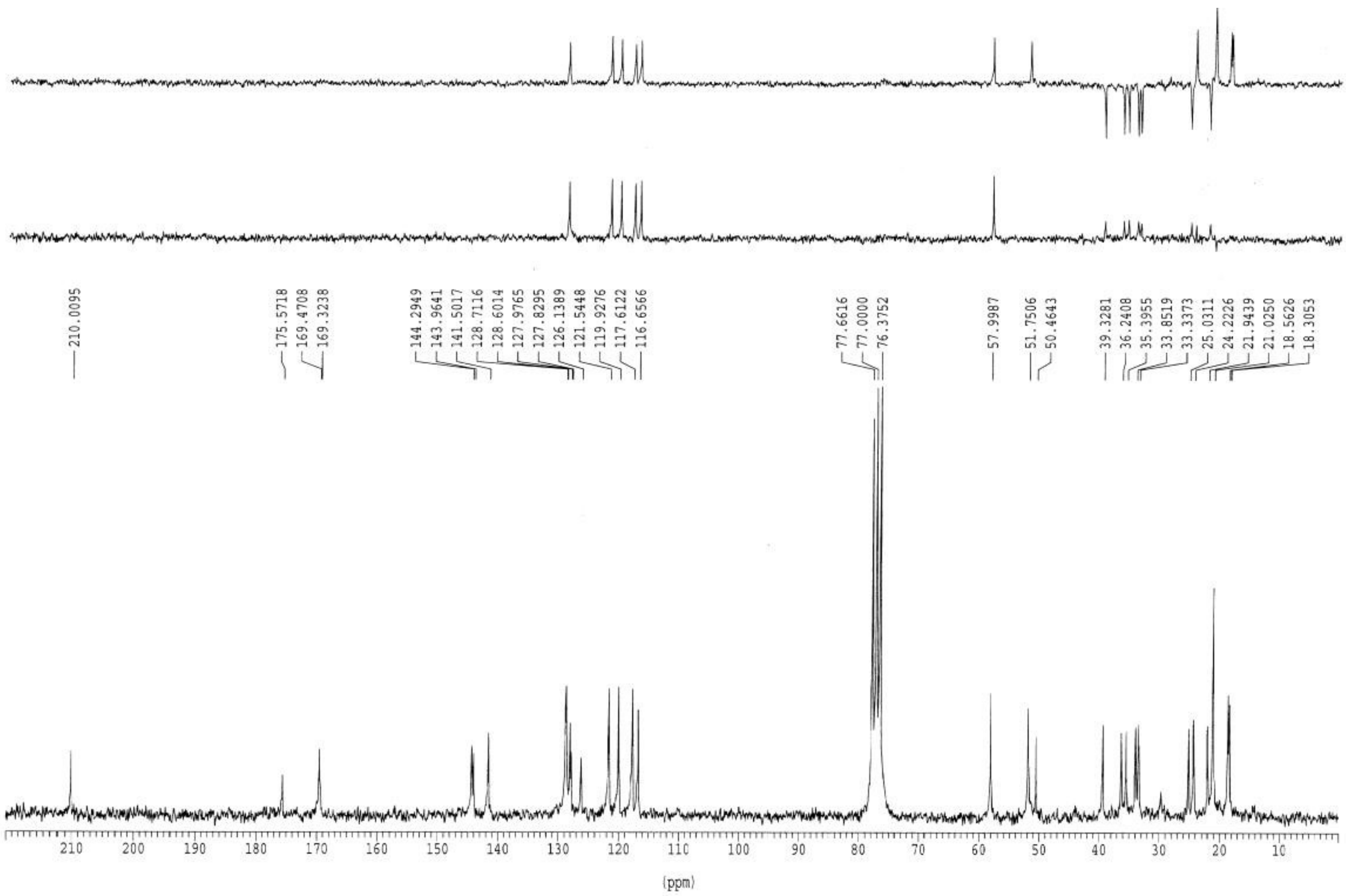

Figure S9: ^{1}H and ^{13}C NMR spectra for compound **10**.

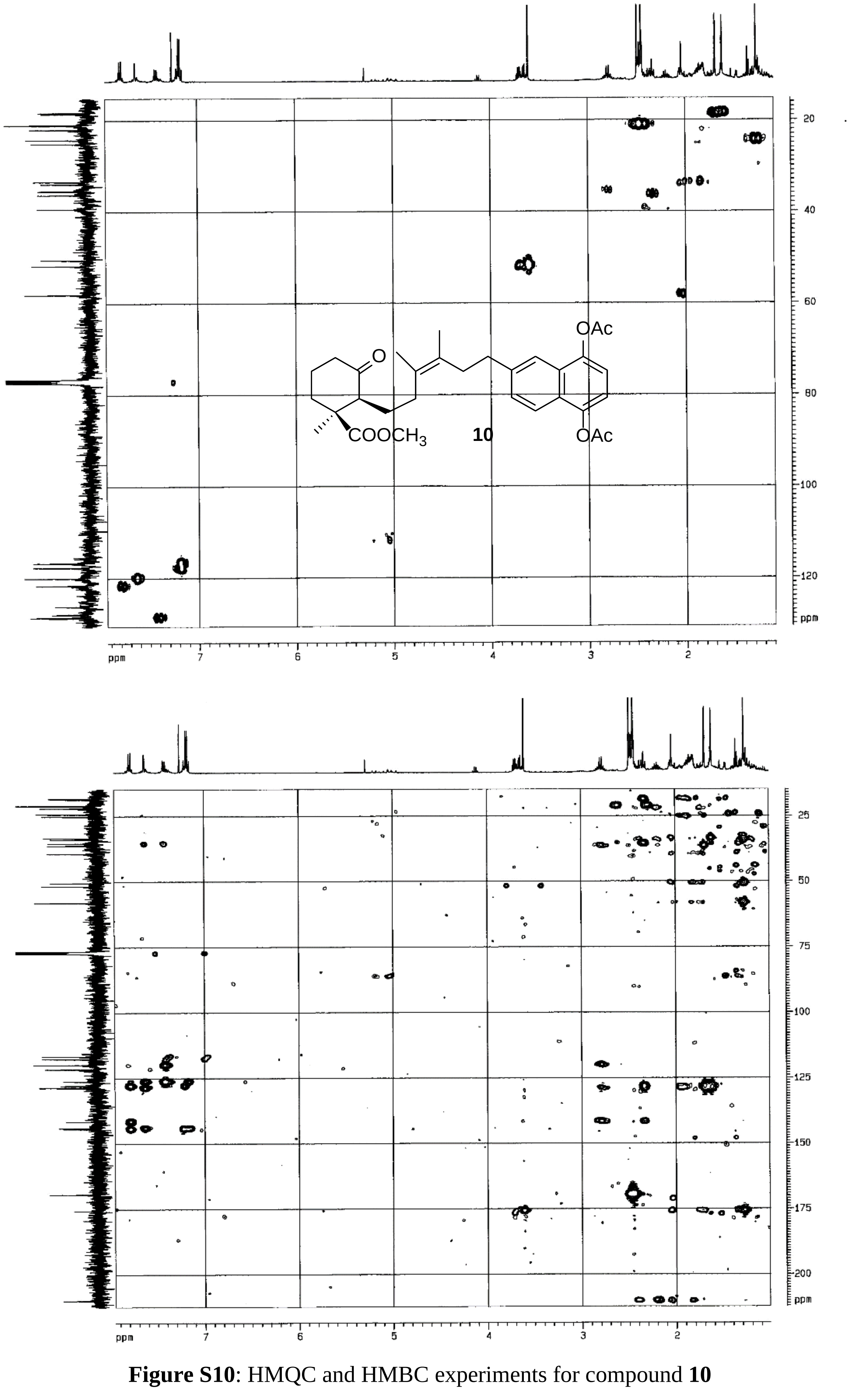

Figure S10: HMQC and HMBC experiments for compound **10**

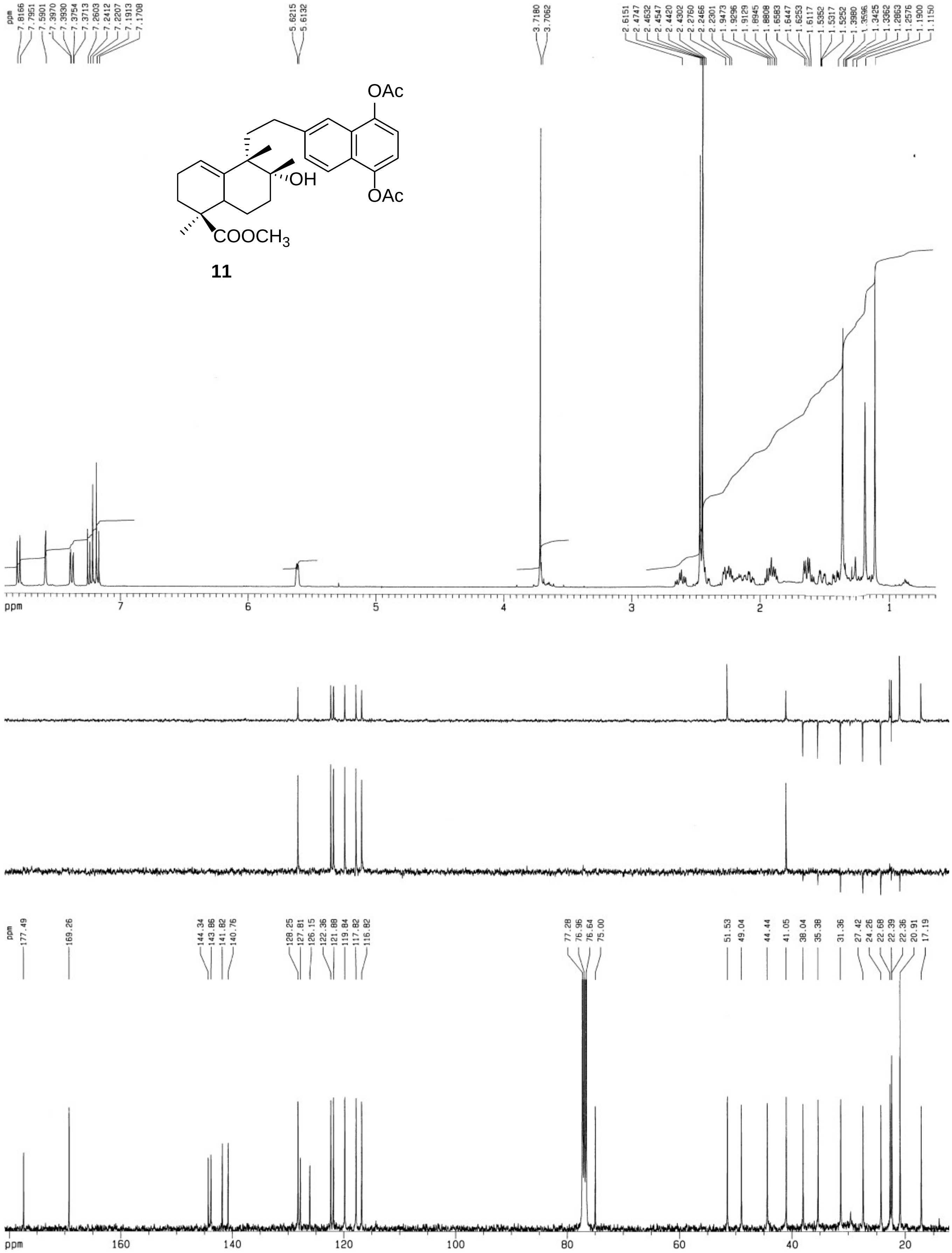

Figure S11: ^{1}H and ^{13}C NMR spectra for compound **11**.

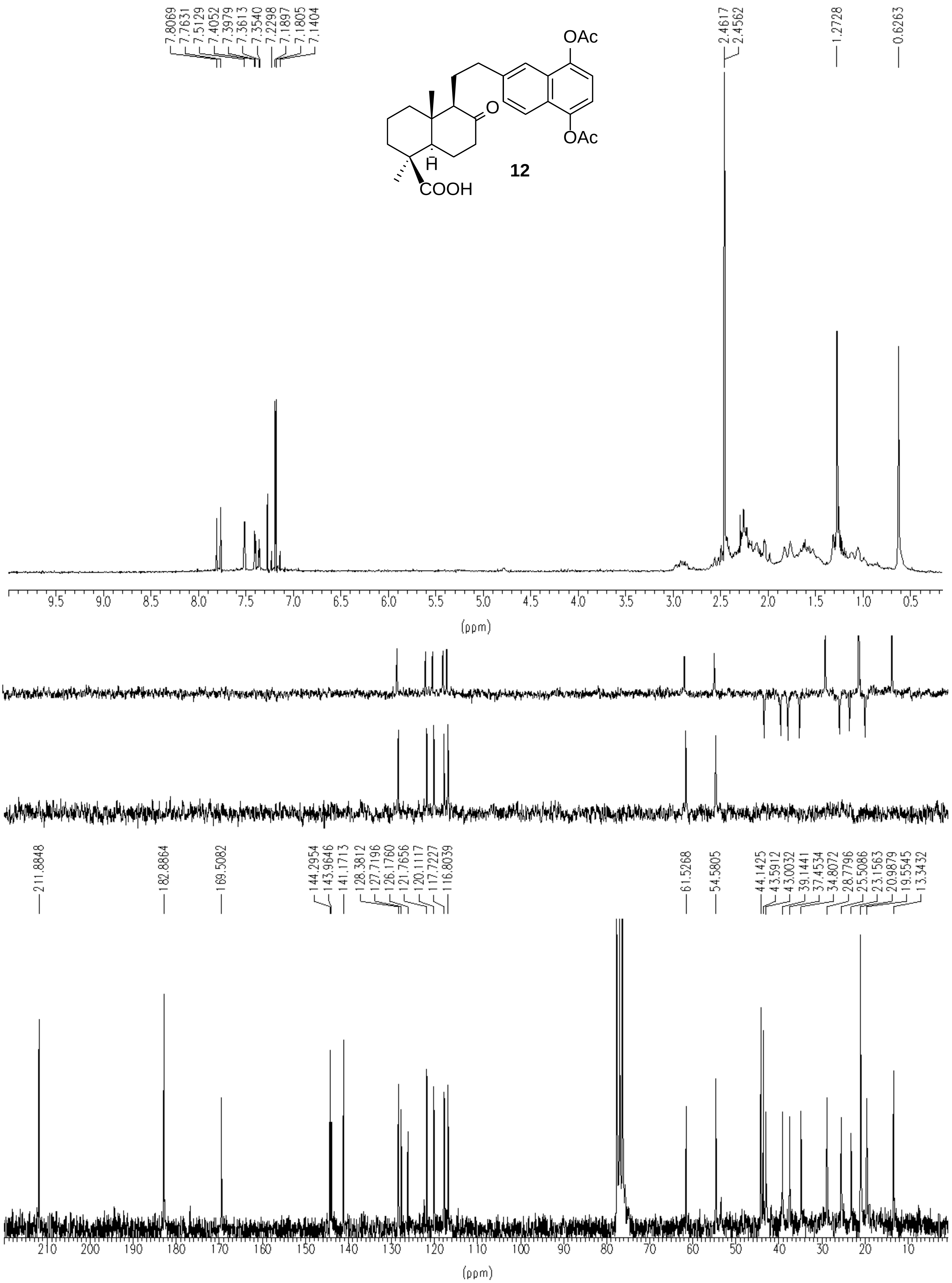

Figure S12:^{1}H and ^{13}C NMR spectra for compound **12**.

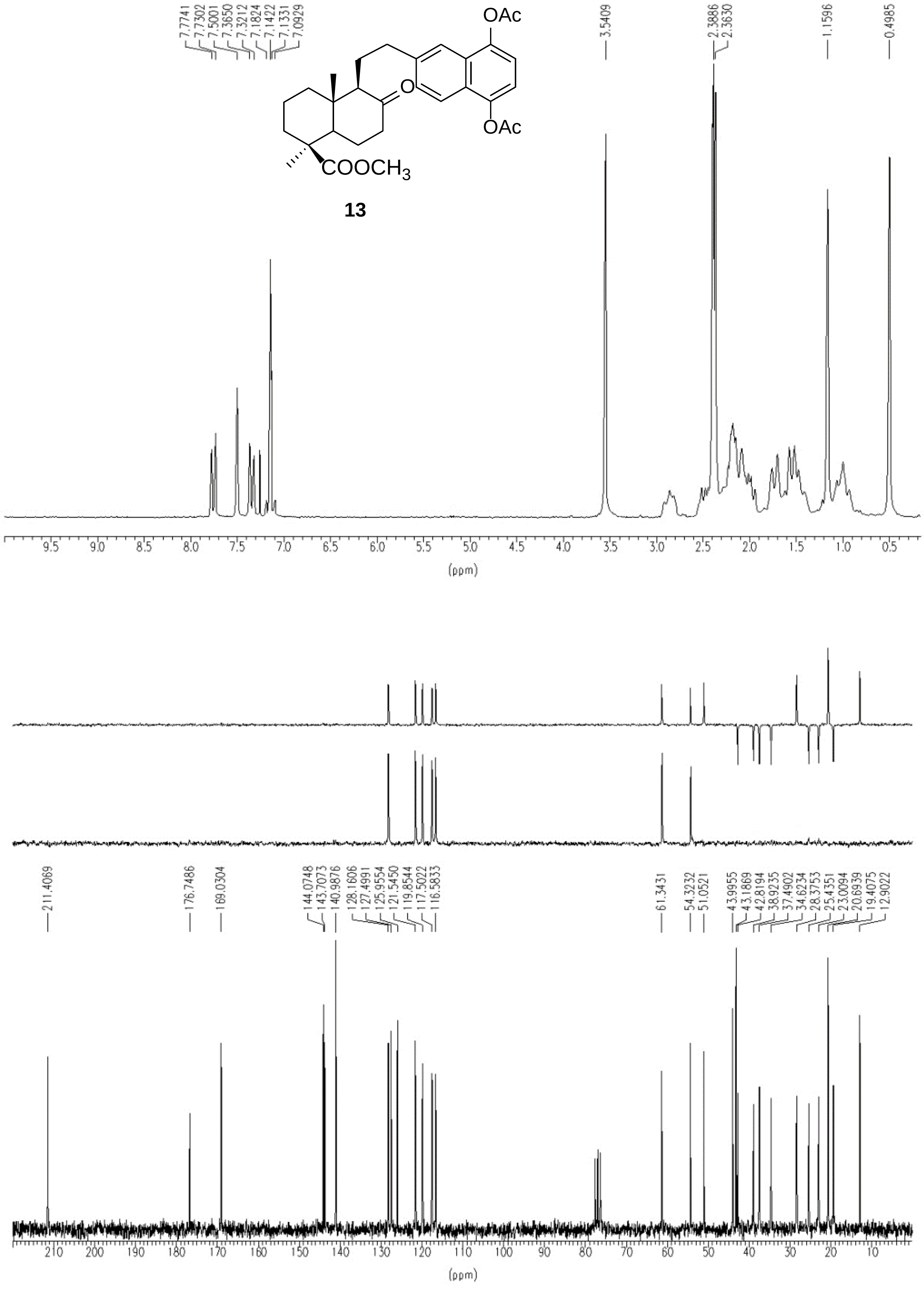

Figure S13: ^{1}H and ^{13}C NMR spectra for compound **13**.

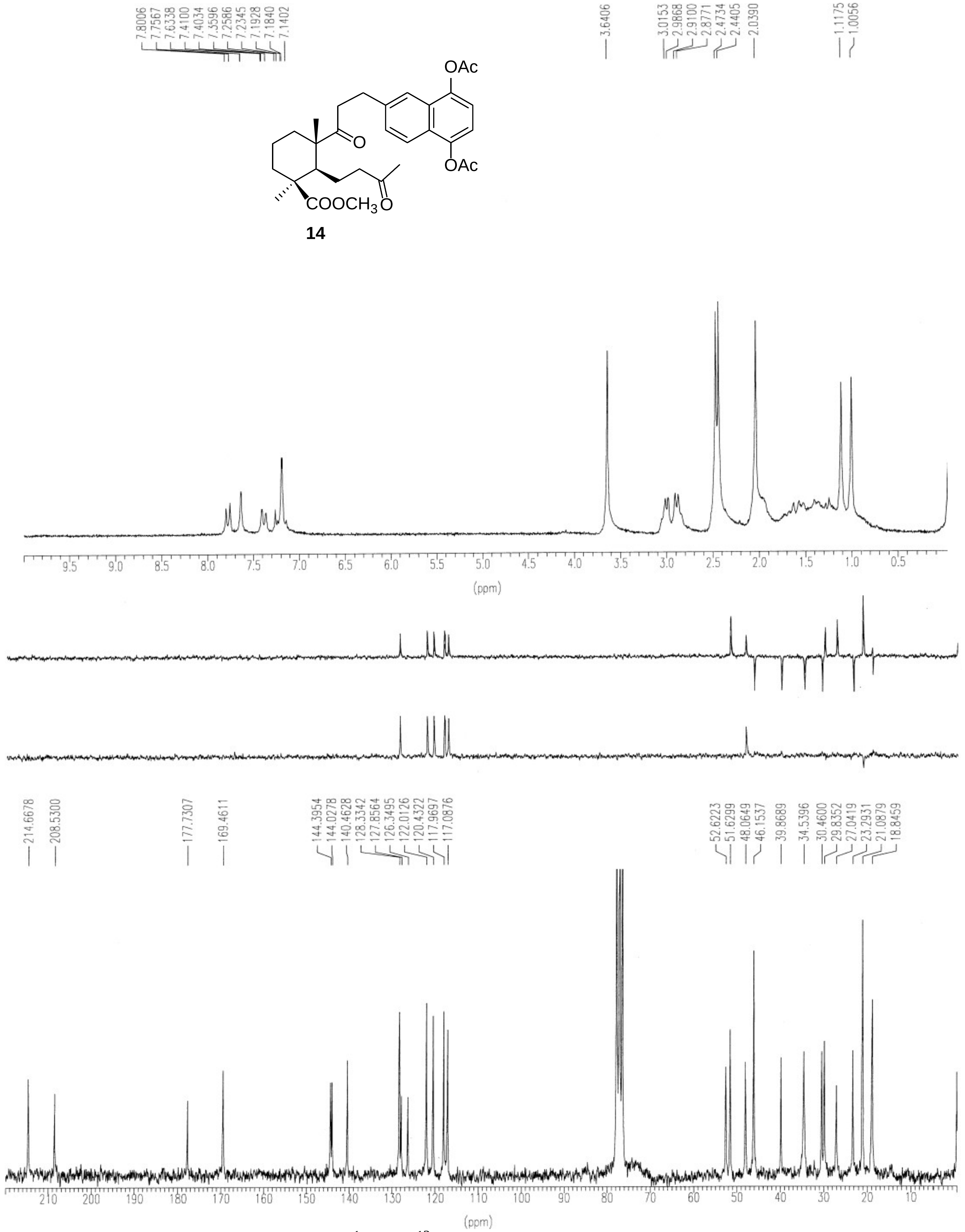

Figure S14:^{1}H and ^{13}C NMR spectra for compound **14**.

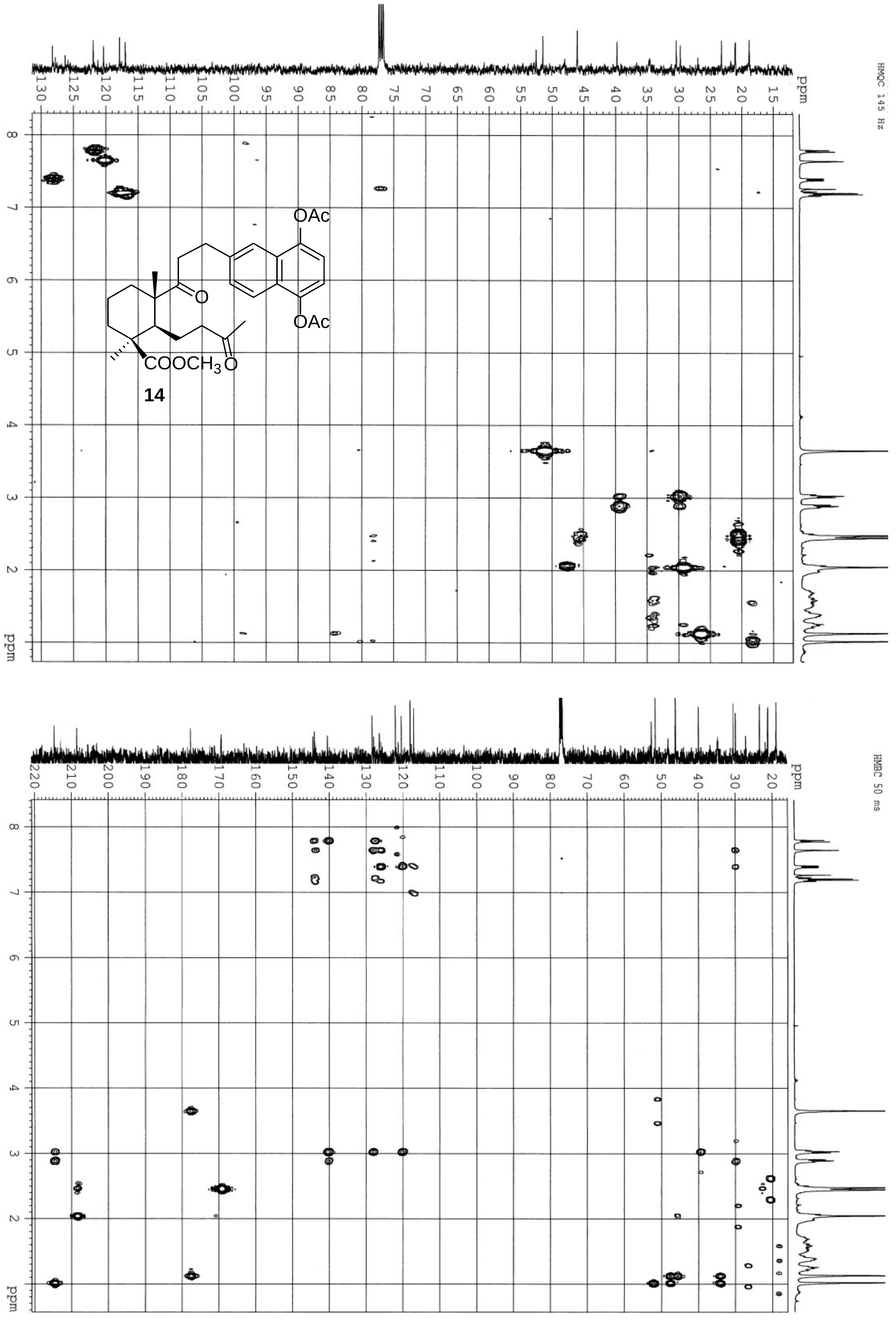

Figure S15: HMQC and HMBC experiments for compound **14.**

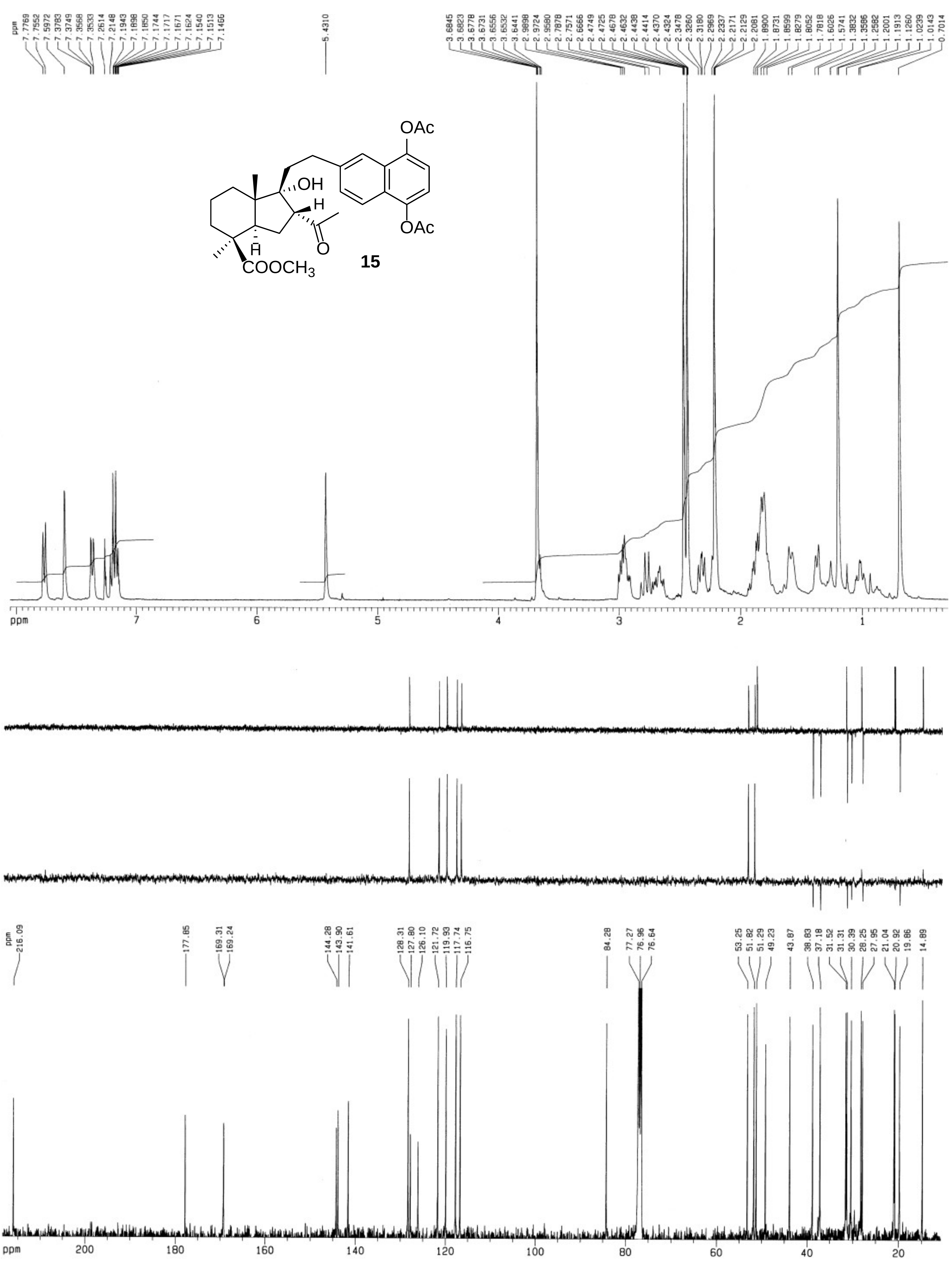

Figure S16: ¹H and ¹³C NMR spectra for compound **15**.

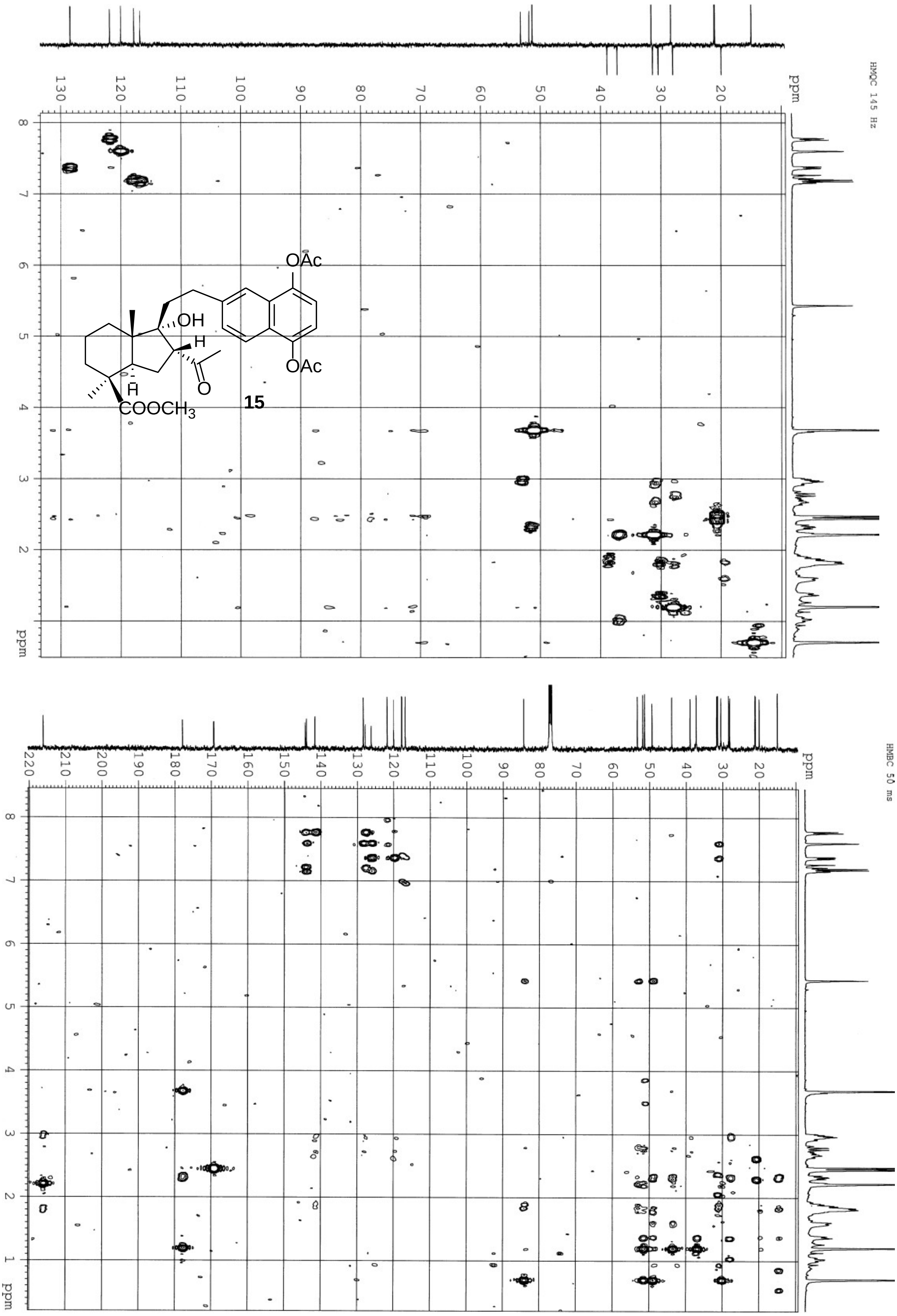

Figure S17: HMQC and HMBC experiments for compound **15.**

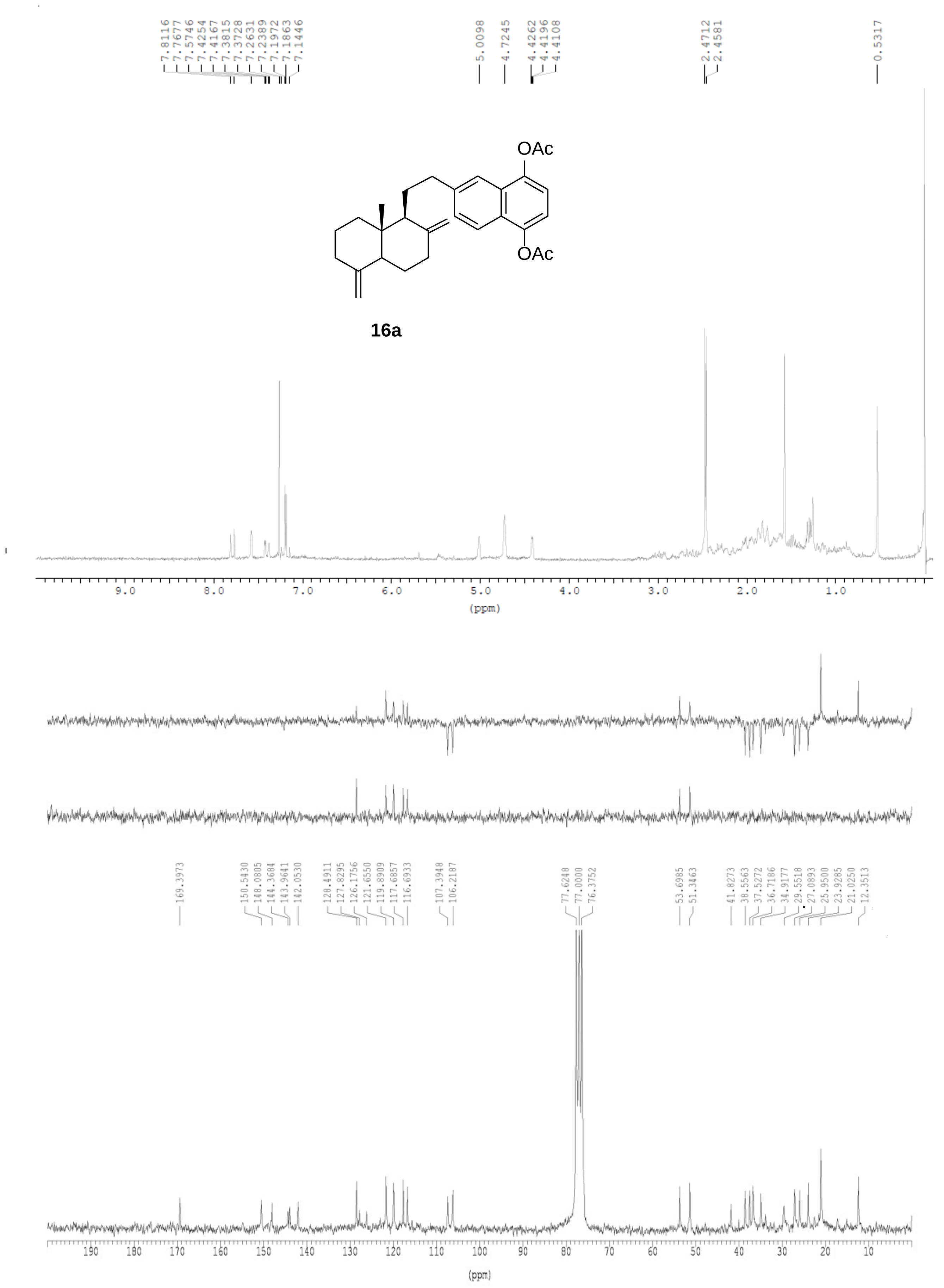

Figure S18: ^{1}H and ^{13}C NMR spectra for compound **16a**.

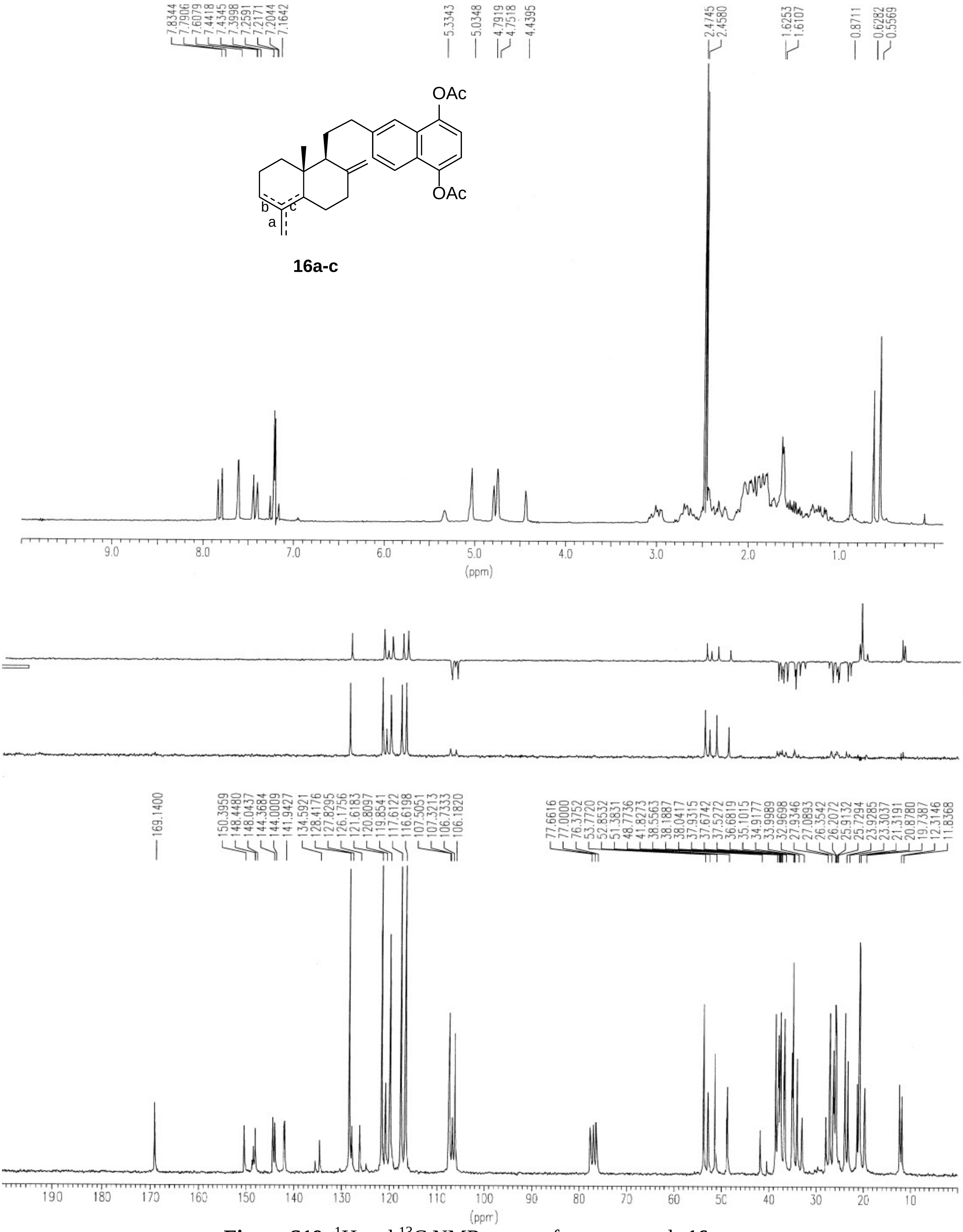

Figure S19: ^{1}H and ^{13}C NMR spectra for compounds **16a-c**.

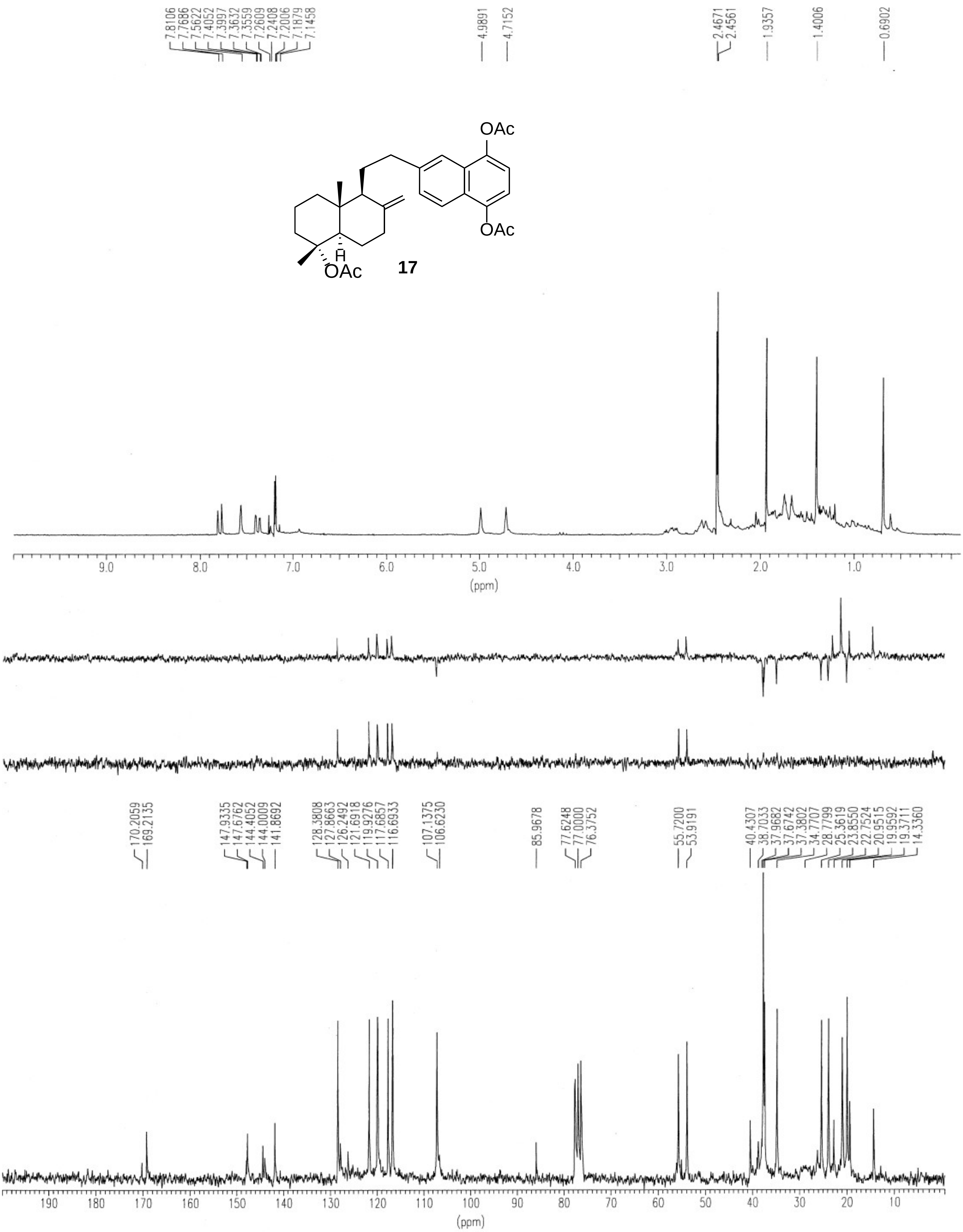

Figure S20: ^{1}H and ^{13}C NMR spectra for compound **17**.

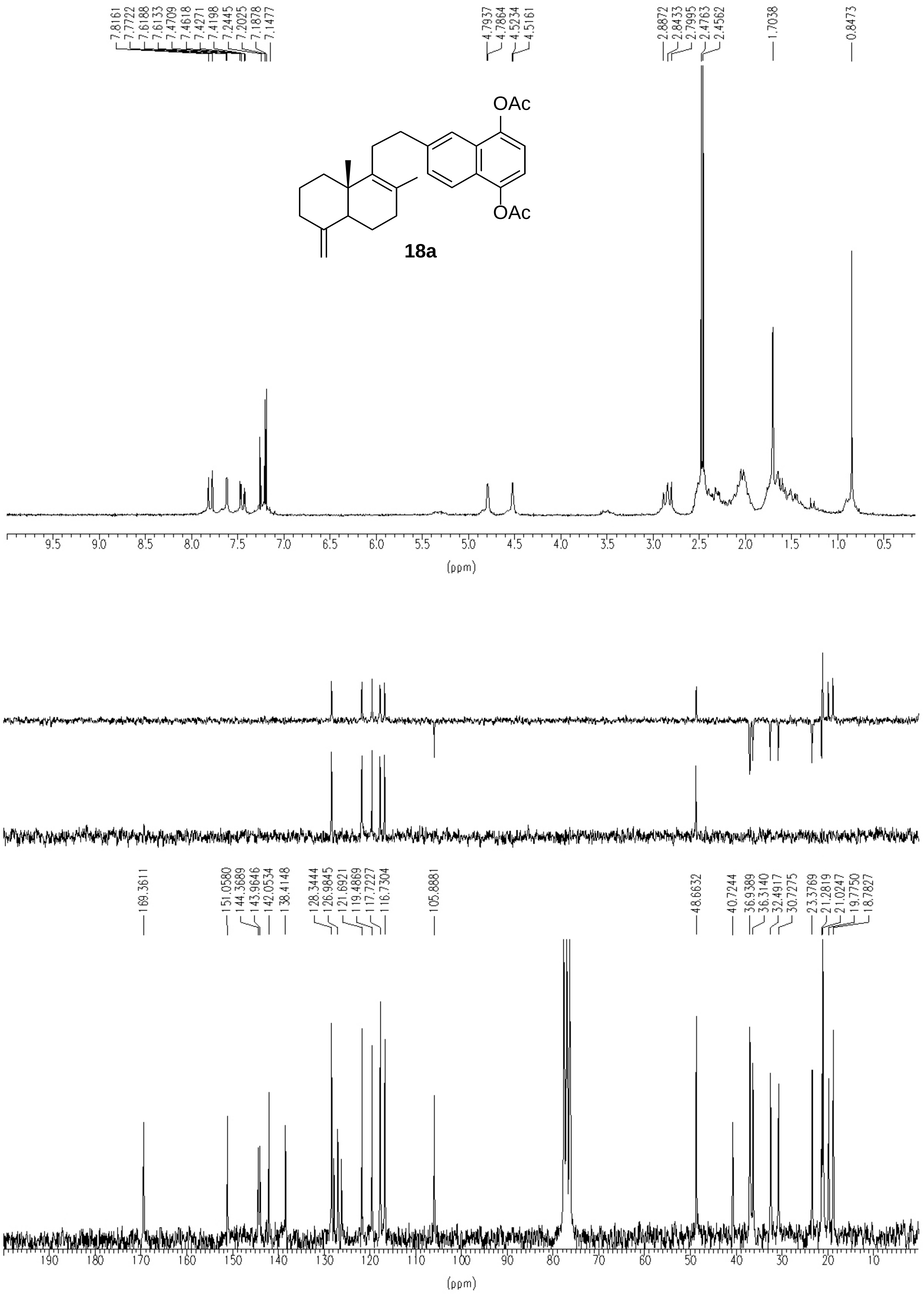

Figure S21: ^{1}H and ^{13}C NMR spectra for compound **18a**.

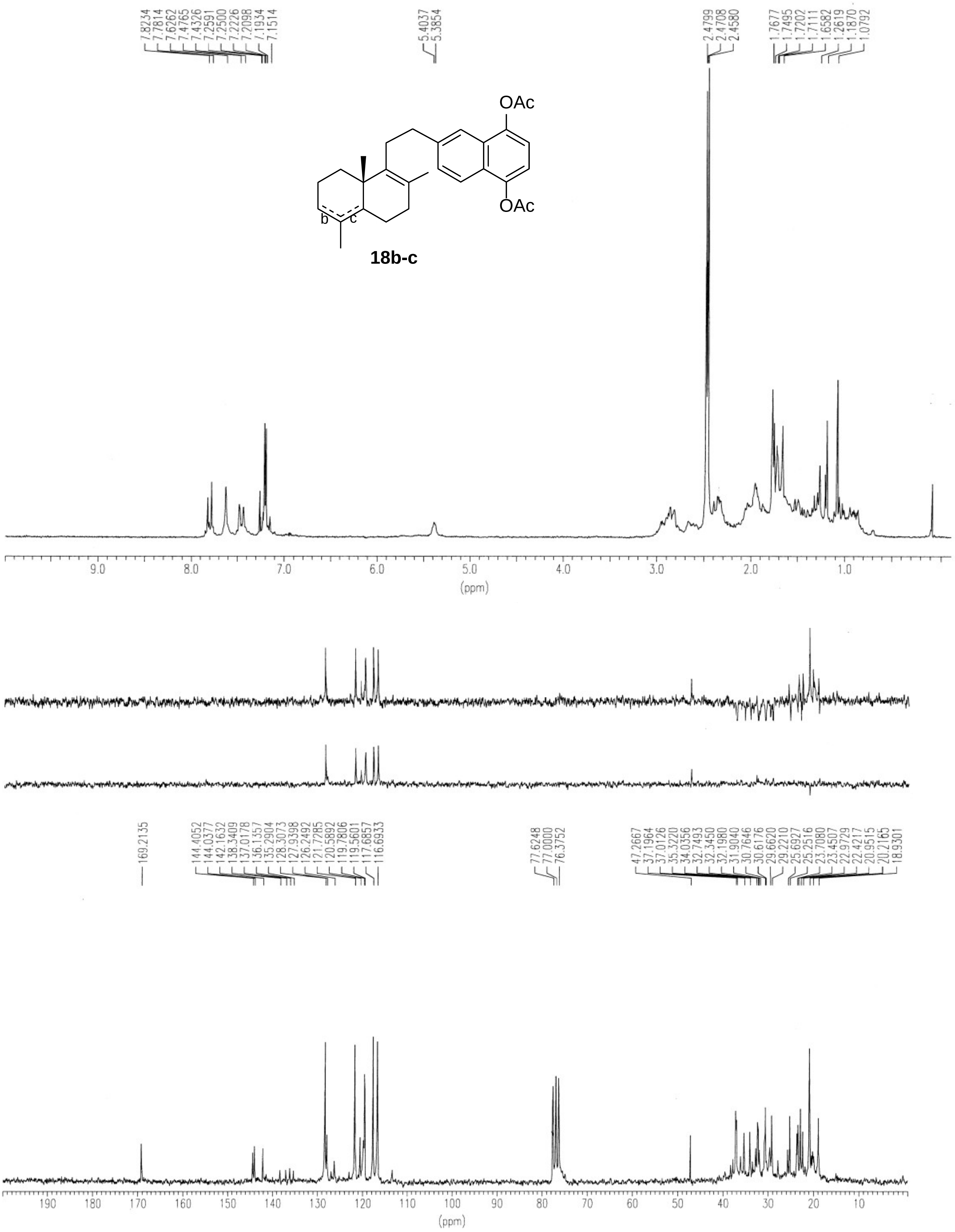

Figure S22: ^{1}H and ^{13}C NMR spectra for compounds **18b** and **18c**.

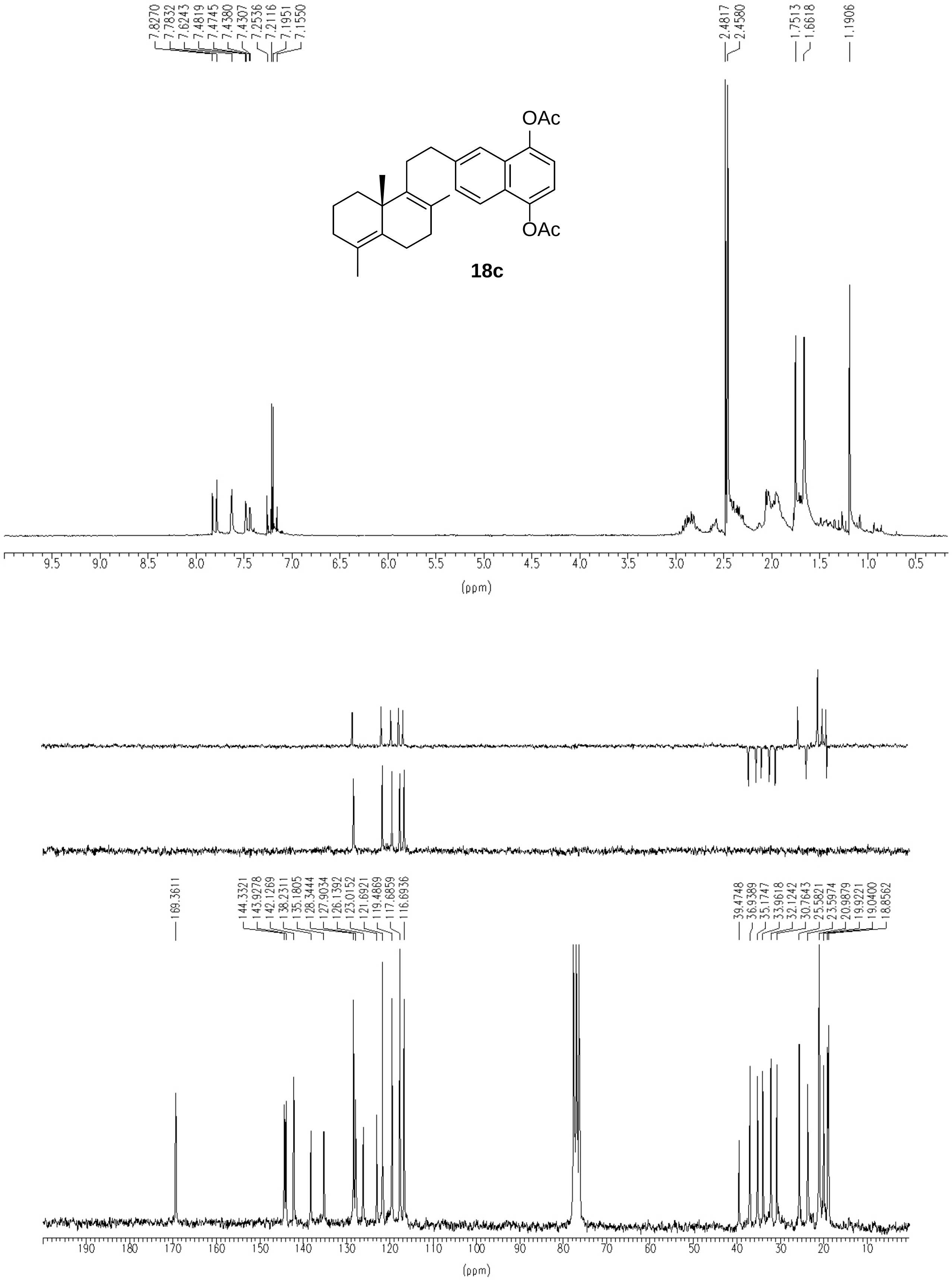

Figure S23: ^{1}H and ^{13}C NMR spectra for compound **18c**.

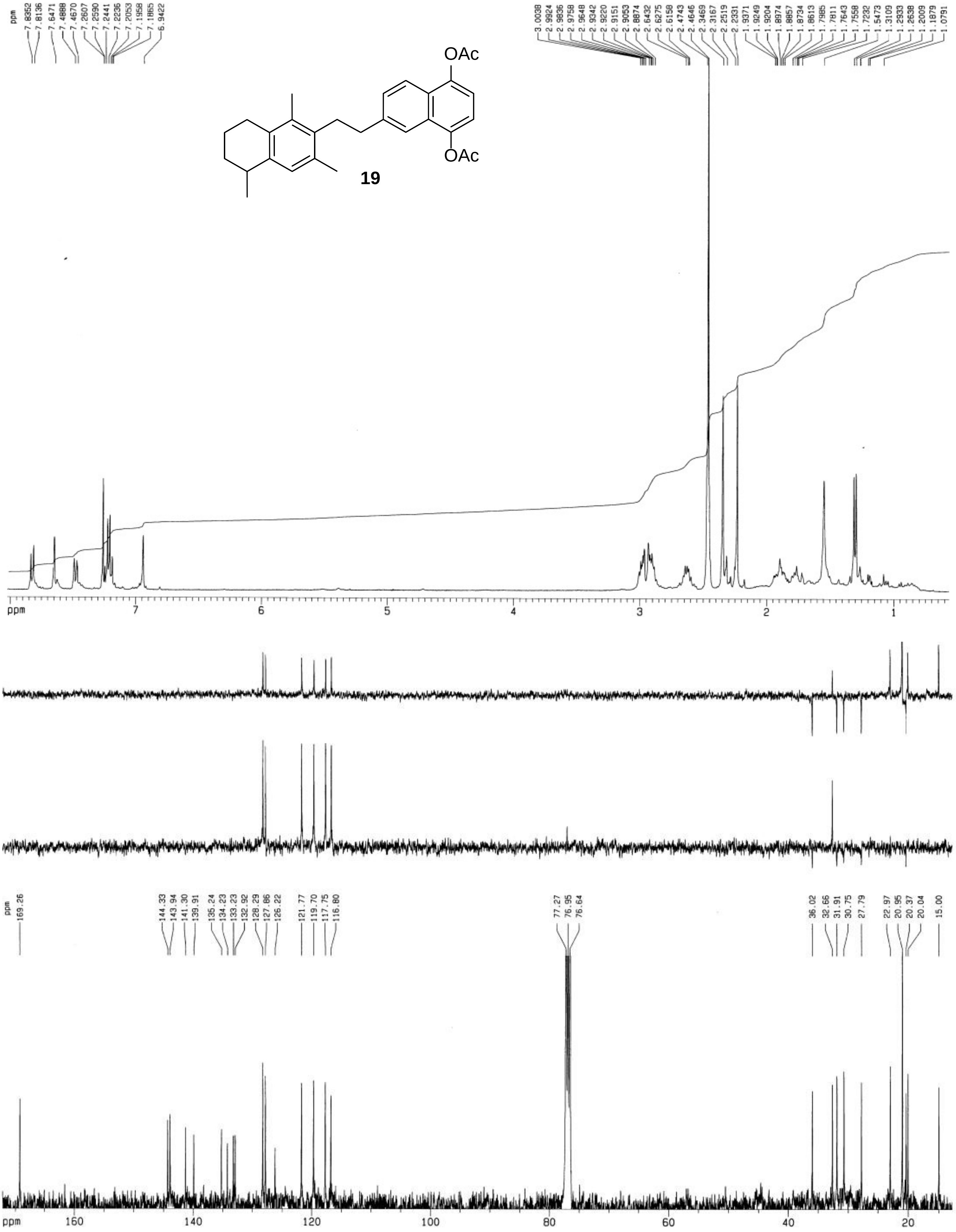

Figure S24: ^{1}H and ^{13}C NMR spectra for compounds **19**.

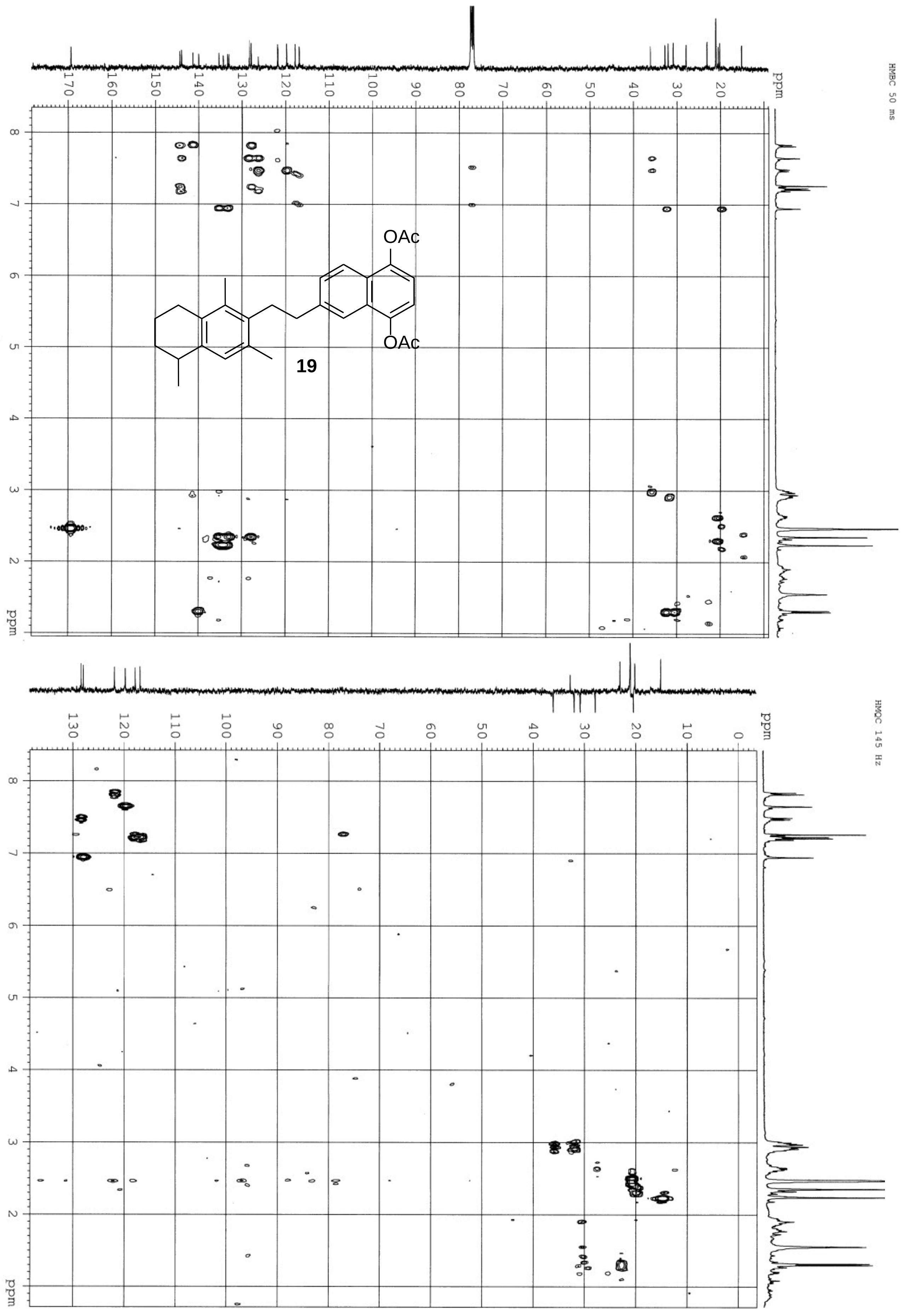

Figure S25: HMQC and HMBC experiments for compound **19**.

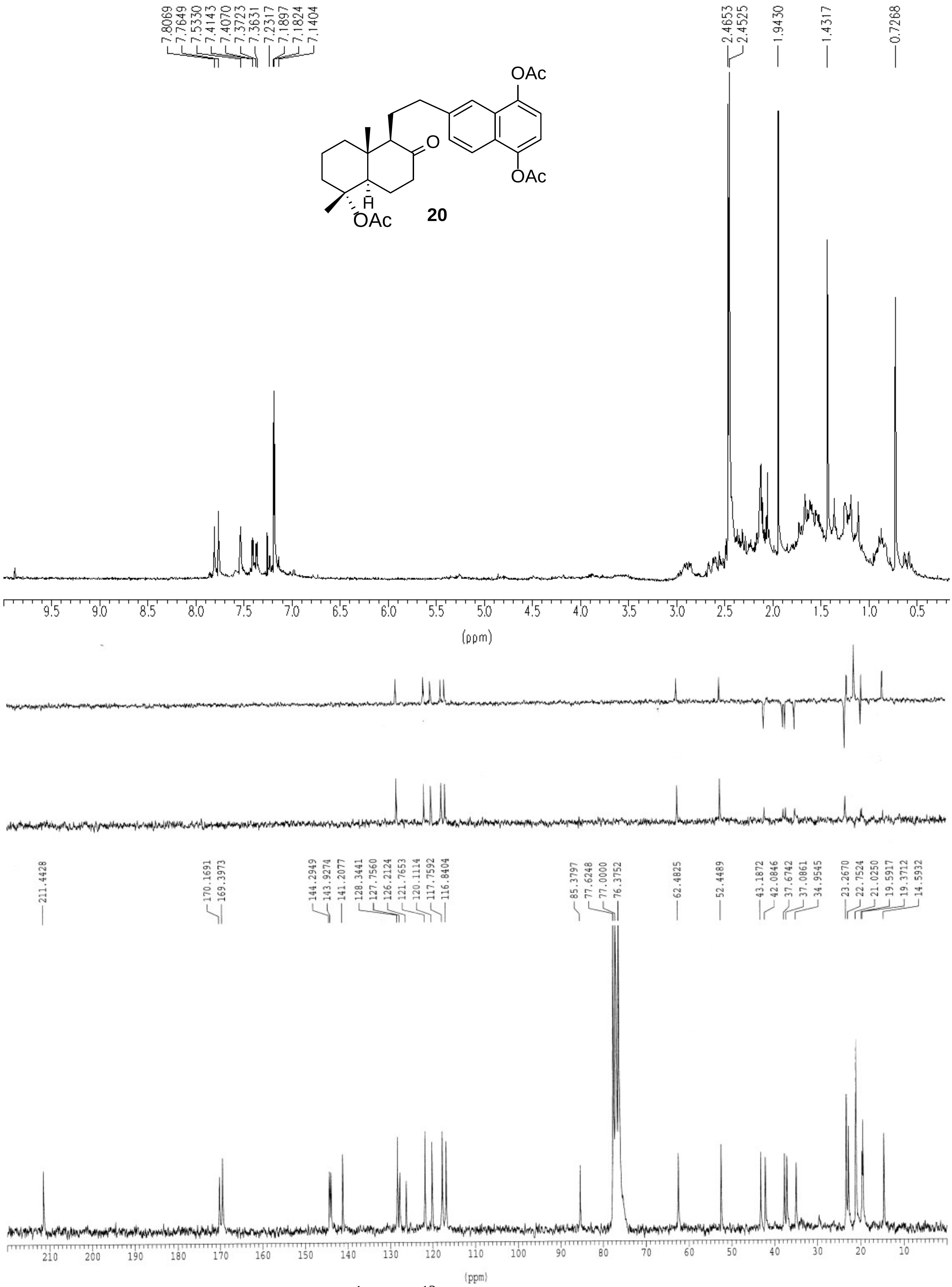

Figure S26: IR, ^{1}H and ^{13}C NMR spectra for compounds **20**.

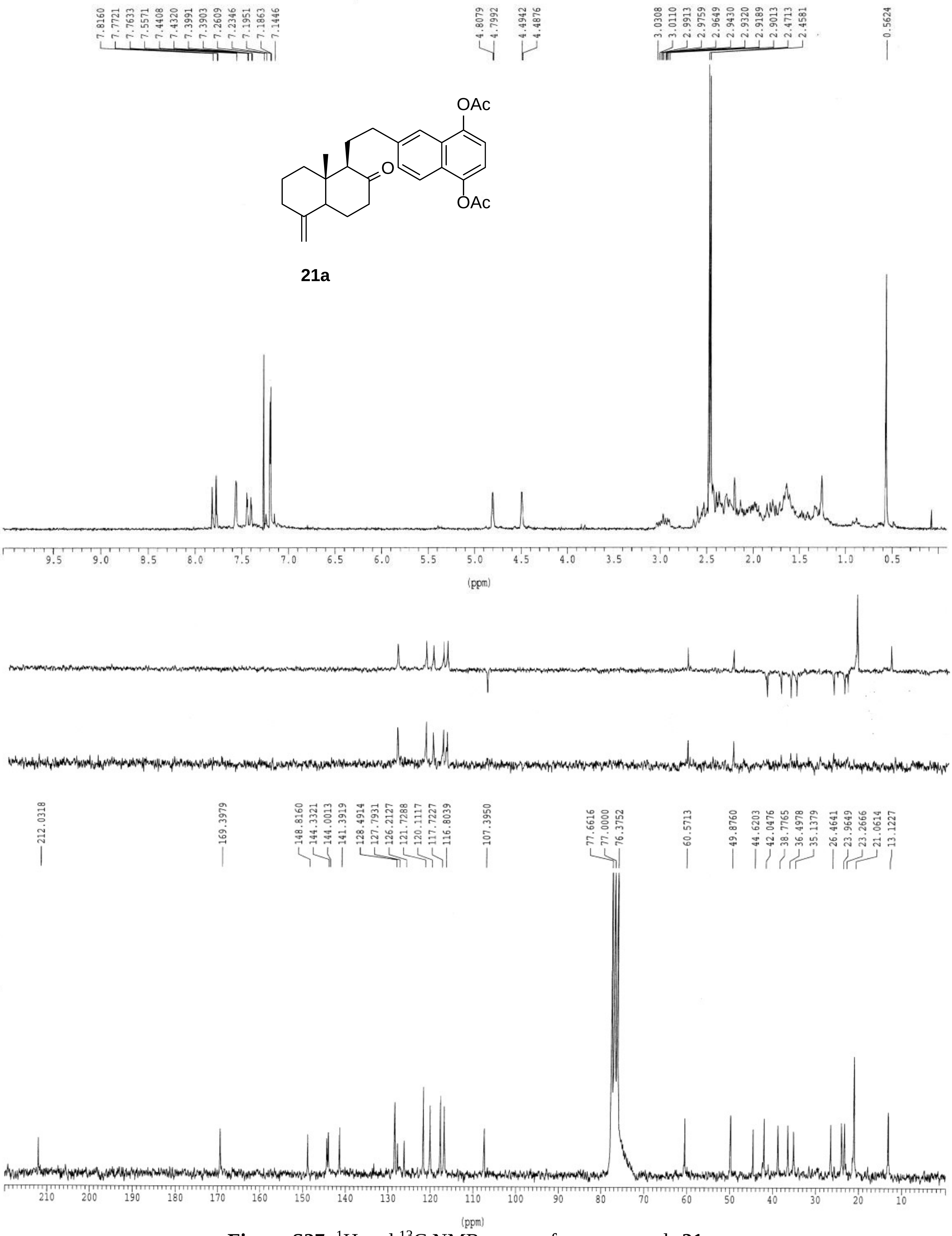

Figure S27: ^{1}H and ^{13}C NMR spectra for compounds **21a**.

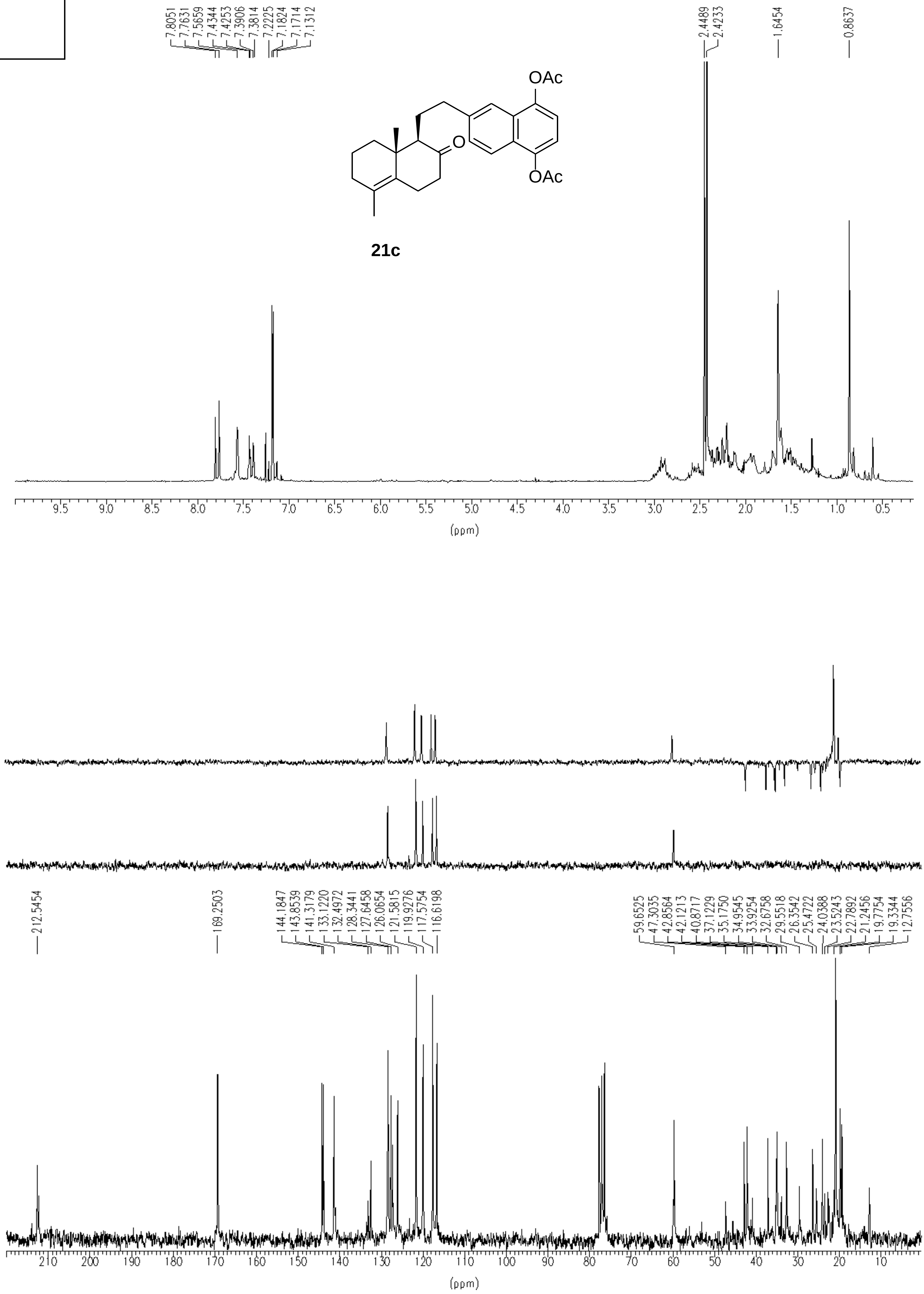

Figure S28: 1H and 13C NMR spectra for compounds **21c**.
